# Supplementary material for: Reconstruction of the cell pseudo-space from single-cell RNA sequencing data with scSpace
Source: Nat Commun. 2023 Apr 29;14:2484. doi: 10.1038/s41467-023-38121-4 (PMC10148590; doi:10.1038/s41467-023-38121-4)
Supplement: Supplementary file 1 — Supplementary Information [file 41467_2023_38121_MOESM1_ESM.pdf]

Reconstruction of the cell pseudo-space from single-cell RNA sequencing data with scSpace

Jingyang Qian†, Jie Liao†, *, Ziqi Liu†, Ying Chi†, Yin Fang, Yanrong Zheng, Xin Shao, Bingqi Liu, Yongjin Cui, Wenbo Guo, Yining Hu, Hudong Bao, Penghui Yang, Qian Chen, Mingxiao Li, Bing Zhang* & Xiaohui Fan*

Supplementary Figures

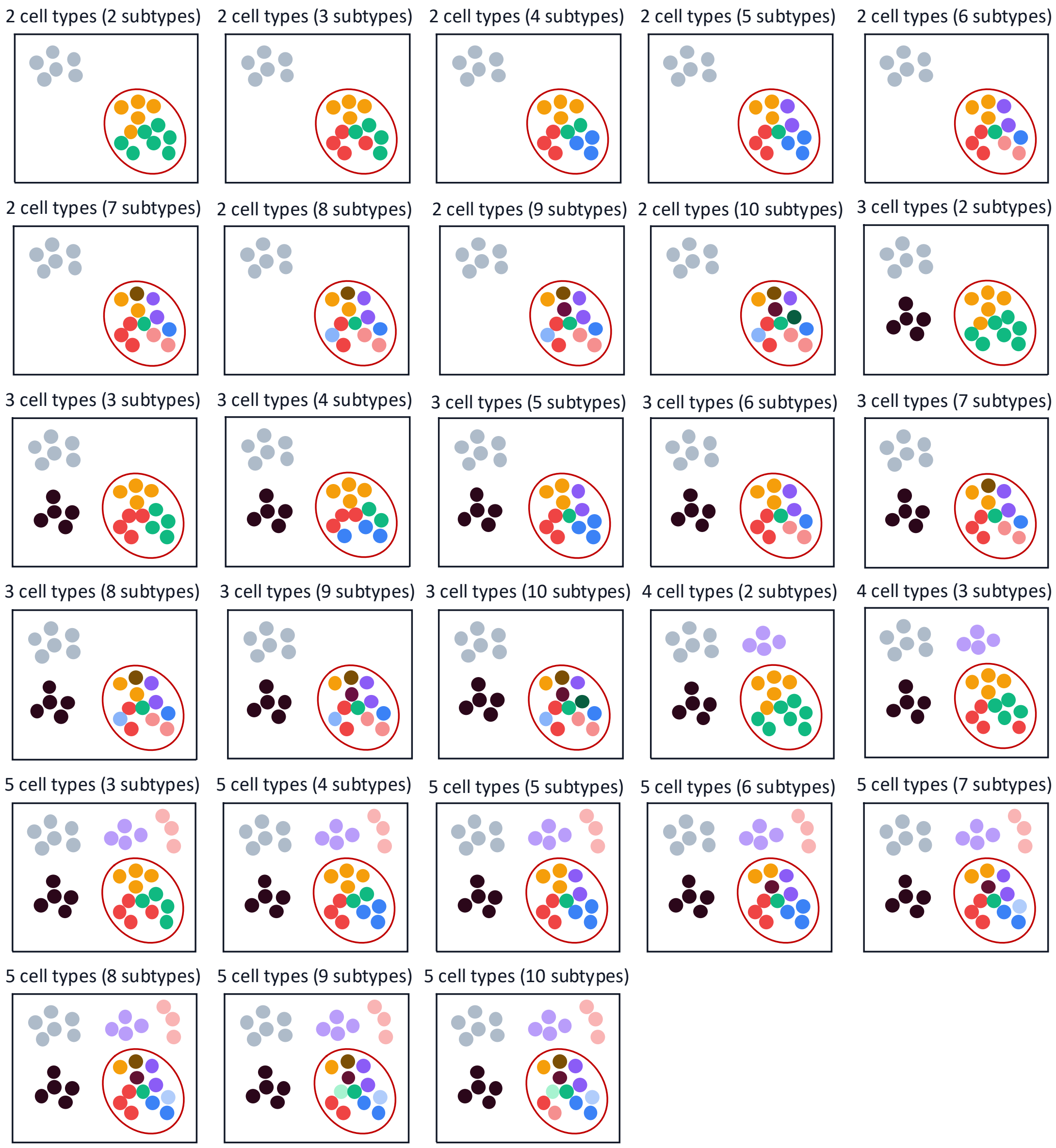

Supplementary Fig. 1. Illustration of the simulated datasets. 140 paired scRNA-seq and spatial transcriptomics data with 2 to 10 spatially heterogeneous subclusters were simulated to evaluate the performance of scSpace.

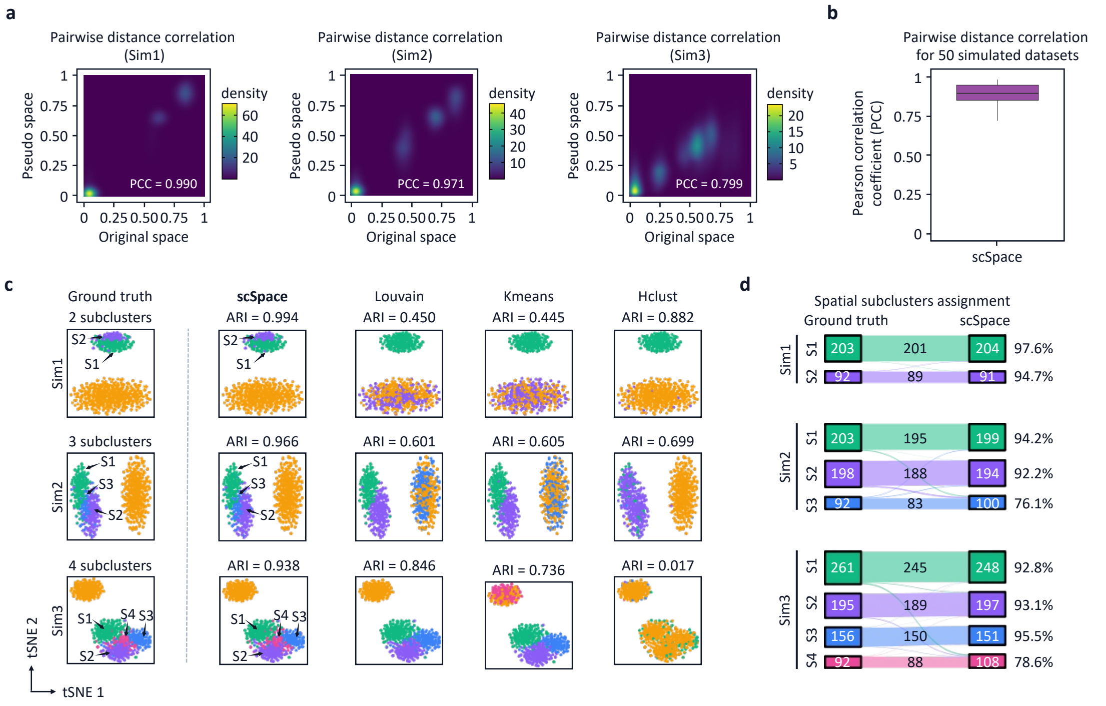

Supplementary Fig. 2. Performance evaluations on simulated datasets. a, Density plot of pairwise distance between cells in the pseudo-space and the original-space for three examples (simulation 1, 2, and 3). **b,** Pairwise distance correlation for 50 simulated datasets (simulation 1-50). Data are presented as the boxplots (minima, 25th percentile, median, 75th percentile, and maxima). **c,** t-SNE visualizations of three simulated scRNA-seq data examples (simulation 1, 2, and 3). Colors: ground truth subcluster labels in simulation and the clustering results of scSpace, Louvain, Kmeans, and Hclust, respectively. **d,** Jaccard index demonstrated the number of cells that were successfully assigned by scSpace to their corresponding subclusters in the ground truth for three examples (simulation 1, 2, and 3).

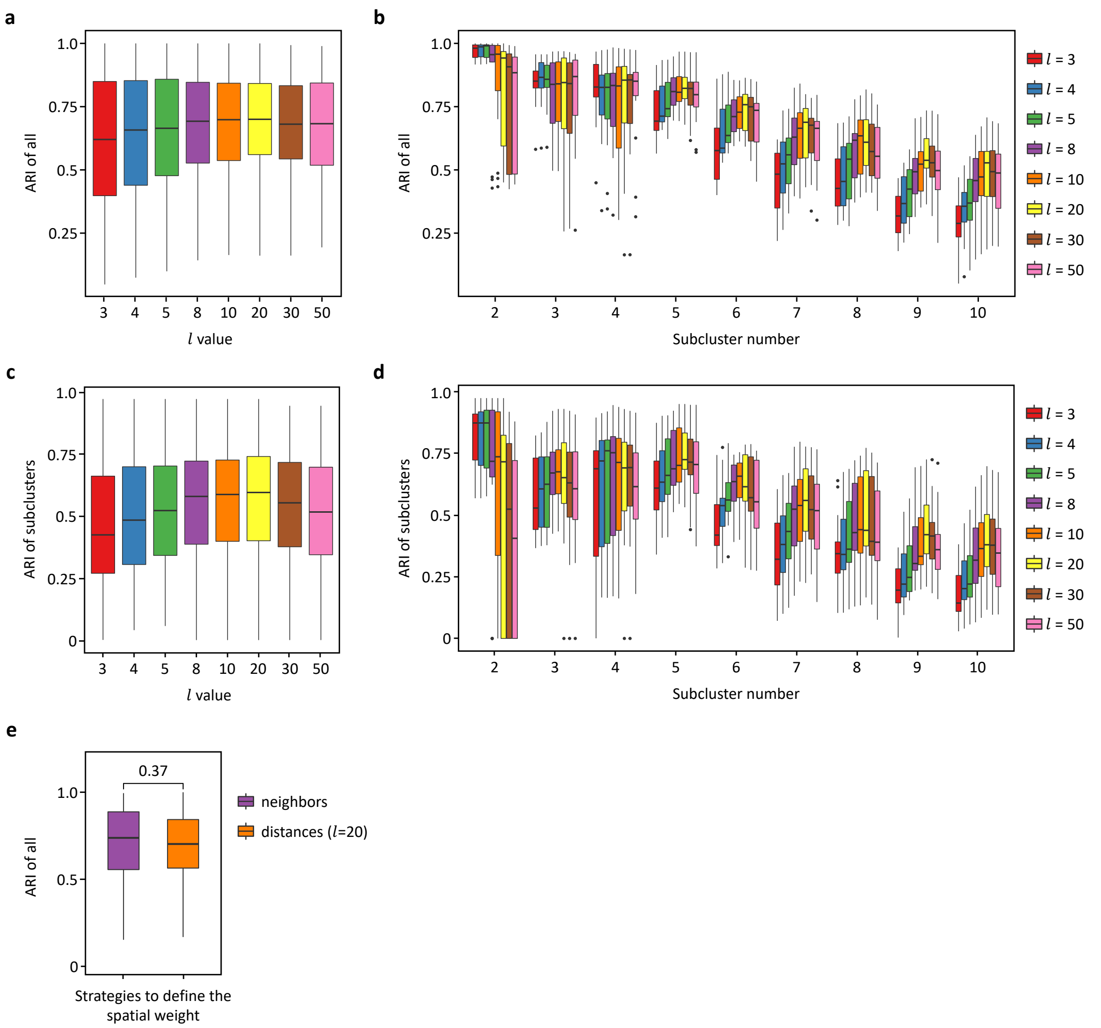

Supplementary Fig. 3. Performance evaluations of two spatial weight construction strategies of scSpace. a, b, ARI score of all clusters under different hyperparameter l setting of scSpace on all 140 simulated datasets (**a**) or simulated datasets with different subcluster number (**b**). Data are presented as the boxplots (minima, 25th percentile, median, 75th percentile, and maxima). **c, d,** ARI score of spatially heterogeneous subclusters under different hyperparameter l setting of scSpace on all 140 simulated datasets (**c**) or simulated datasets with different subcluster number (**d**). Data are presented as the boxplots (minima, 25th percentile, median, 75th percentile, and maxima). **e,** Performance comparison between two spatial weight construction strategies of scSpace using the simulated datasets (n = 140). Data are presented as the boxplots (minima, 25th percentile, median, 75th percentile, and maxima). *P*-value is calculated with the two-sided Wilcoxon rank-sum test.

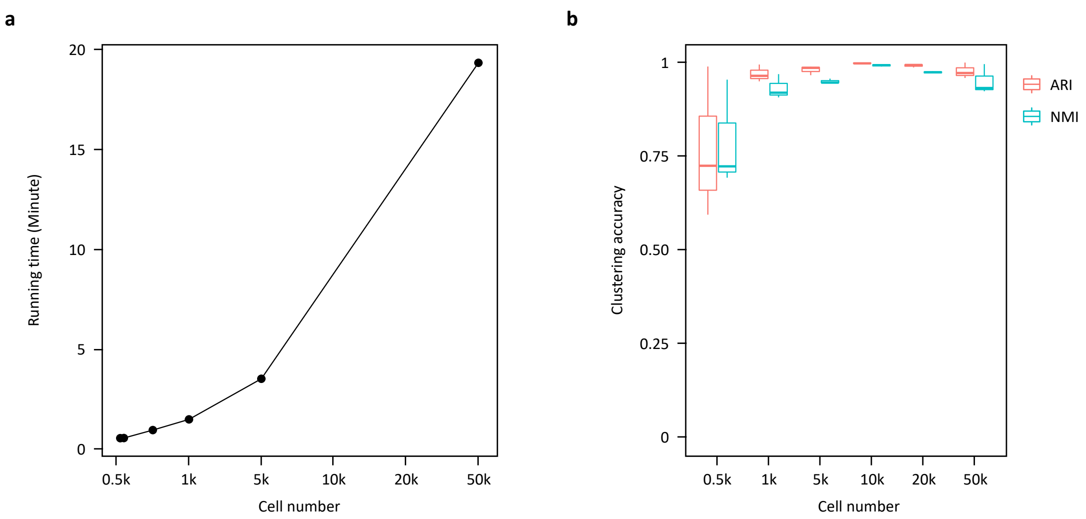

Supplementary Fig. 4. Evaluation of the scalability of scSpace. a, b, The computation time (**a**) and clustering accuracy (**b**) of scSpace evaluated by using the simulated datasets (n = 3). Data are presented as the boxplots (minima, 25th percentile, median, 75th percentile, and maxima).

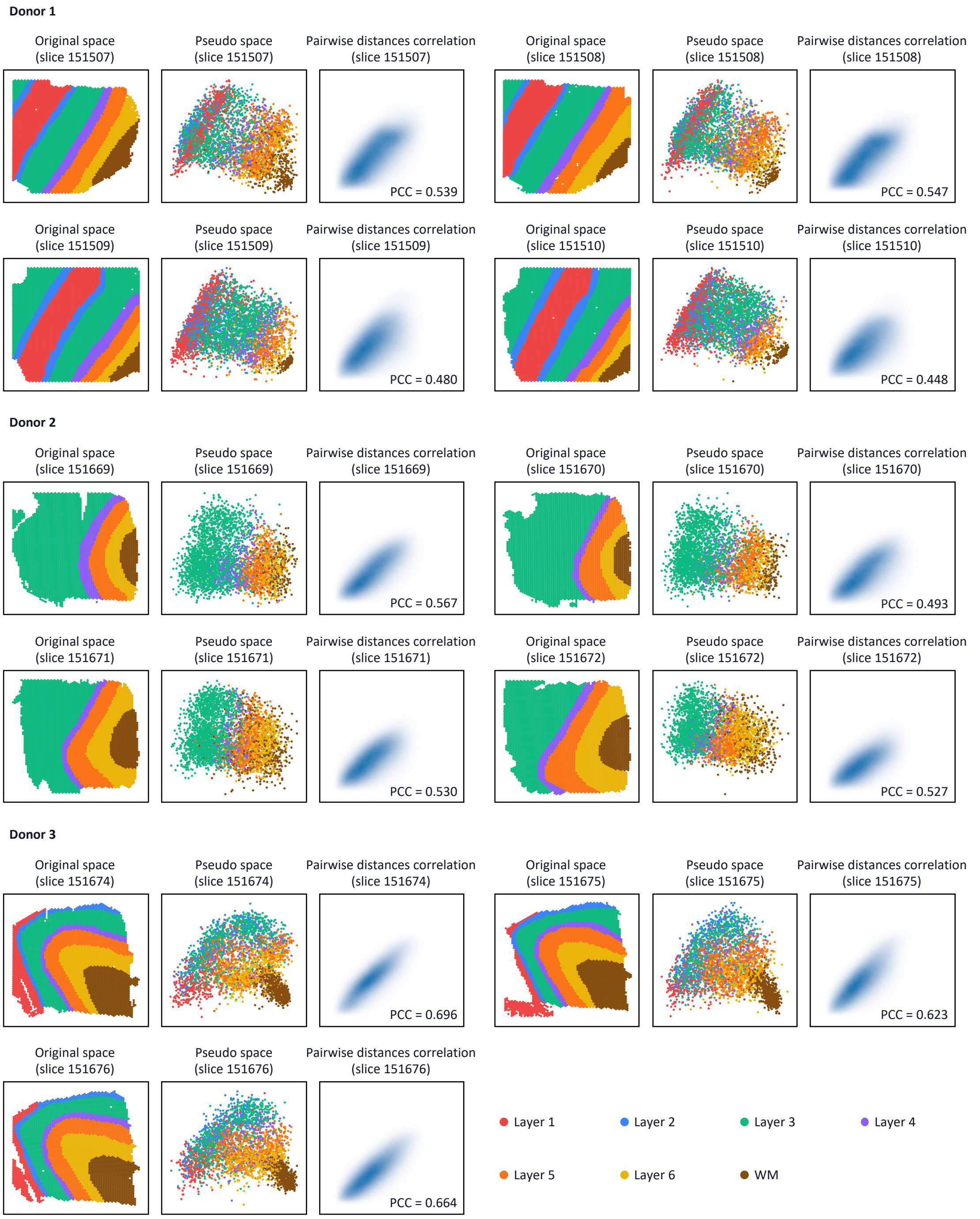

Supplementary Fig. 5. Reconstructing the hierarchical structure of human dorsolateral prefrontal cortex (DLPFC) using 10x Visium spatial transcriptomics data. Extra 11 examples (slice 151507, 151508, 151509, 151510, 151669, 151670, 151671, 151672, 151674, 151675, and 151676) in addition to Figure 2 in the manuscript were showed.

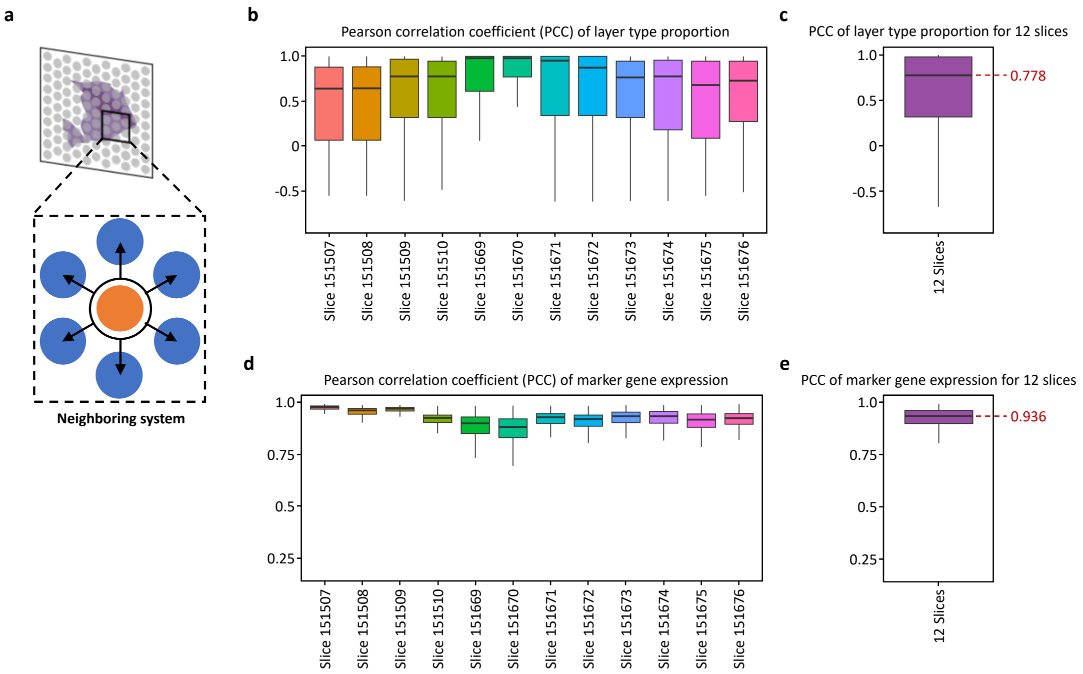

Supplementary Fig. 6. Evaluation the performance of scSpace on human DLPFC data by the neighbor system. a, Illustration of the neighboring system. **b,** Pearson correlation coefficient of layer type proportion for each slice. Data are presented as the boxplots (minima, 25th percentile, median, 75th percentile, and maxima). The number of data points for the boxplots from left to right are 4,221, 4,381, 4,788, 4,595, 3,636, 3,484, 4,093, 3,888, 3,611, 3,635, 3,566, and 3431, respectively. **c,** Pearson correlation coefficient of layer type proportion for 12 slices (n = 47329). Data are presented as the boxplots (minima, 25th percentile, median, 75th percentile, and maxima). **d,** Pearson correlation coefficient of marker gene expression for each slice. Data are presented as the boxplots (minima, 25th percentile, median, 75th percentile, and maxima). The number of data points for the boxplots from left to right are 4,221, 4,381, 4,788, 4,595, 3,636, 3,484, 4,093, 3,888, 3,611, 3,635, 3,566, and 3431, respectively. **e,** Pearson correlation coefficient of marker gene expression for 12 slices (n = 47329). Data are presented as the boxplots (minima, 25th percentile, median, 75th percentile, and maxima).

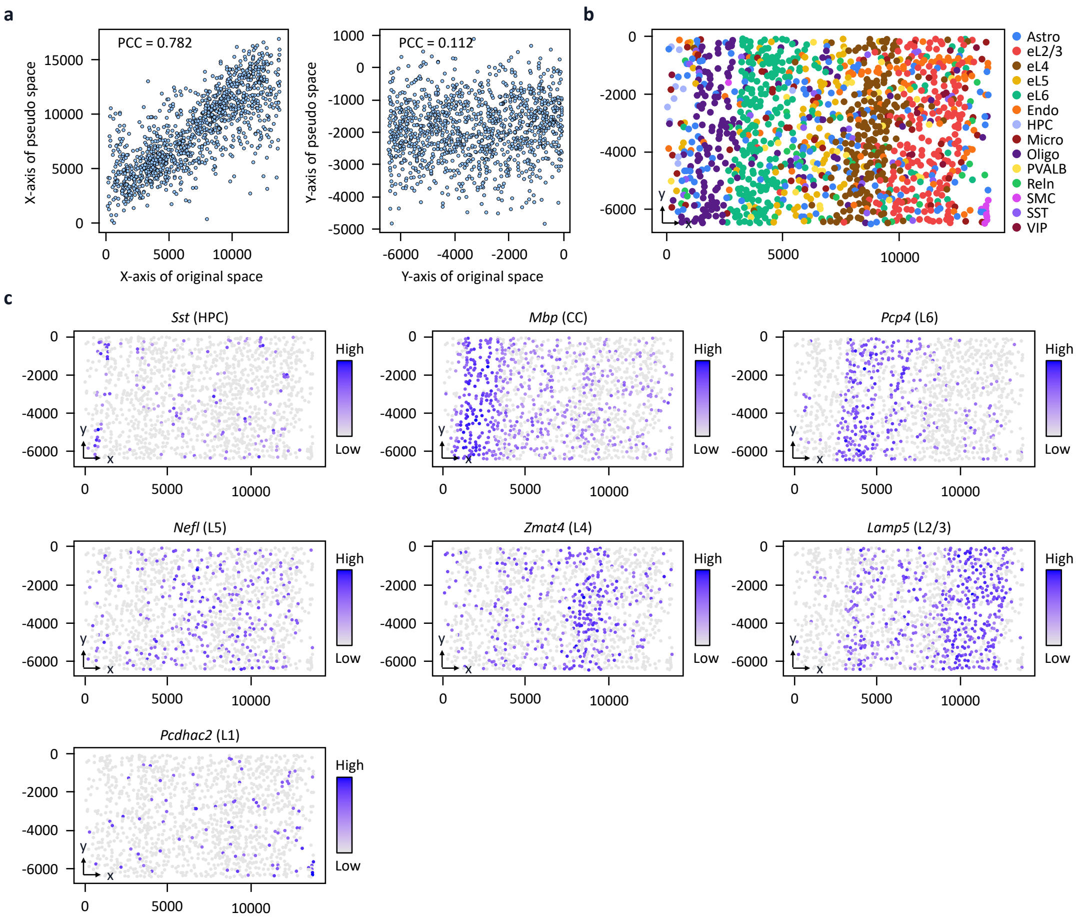

Supplementary Fig. 7. Reconstructing the hierarchical structure of mouse V1 neocortex using STARmap spatial transcriptomics data. a, The performance of spatial reconstruction of scSpace along the X-axis (left) and Y-axis (right) of the tissue slice. **b,** The single cell annotation of STARmap spatial transcriptomics data. Astro, astrocytes; eL2/3, eL4, eL5, eL6, excitatory neuron subtypes; Endo, endothelial cells; HPC, hippocampus; Micro, microglia; Oligo, oligodendrocytes; SMC, smooth muscle cells. **c,** The spatial expression pattern of layer markers.

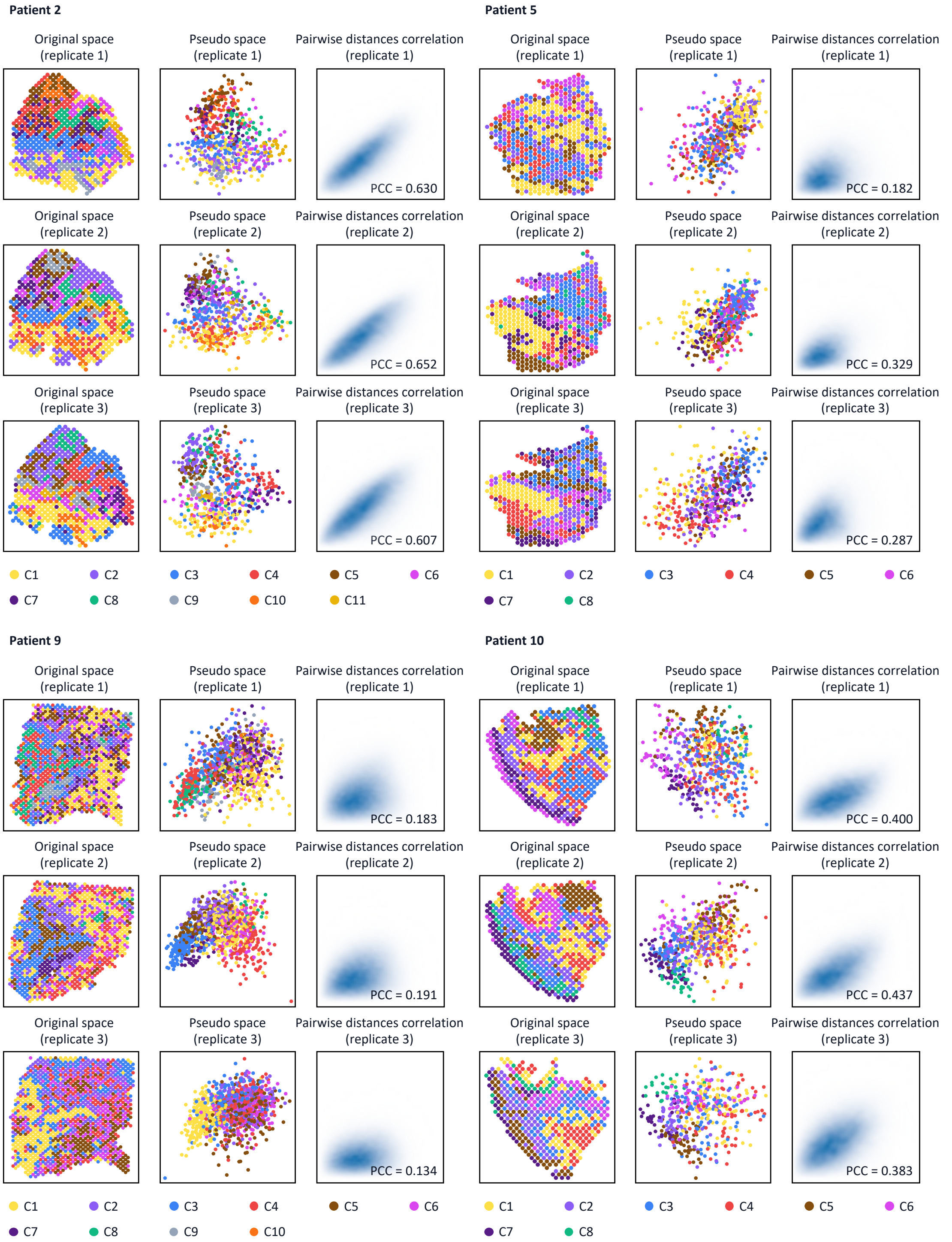

Supplementary Fig. 8. Revealing the shared spatial coherence between replicates of ST data from different patients of the human skin squamous cell carcinoma (SCC).

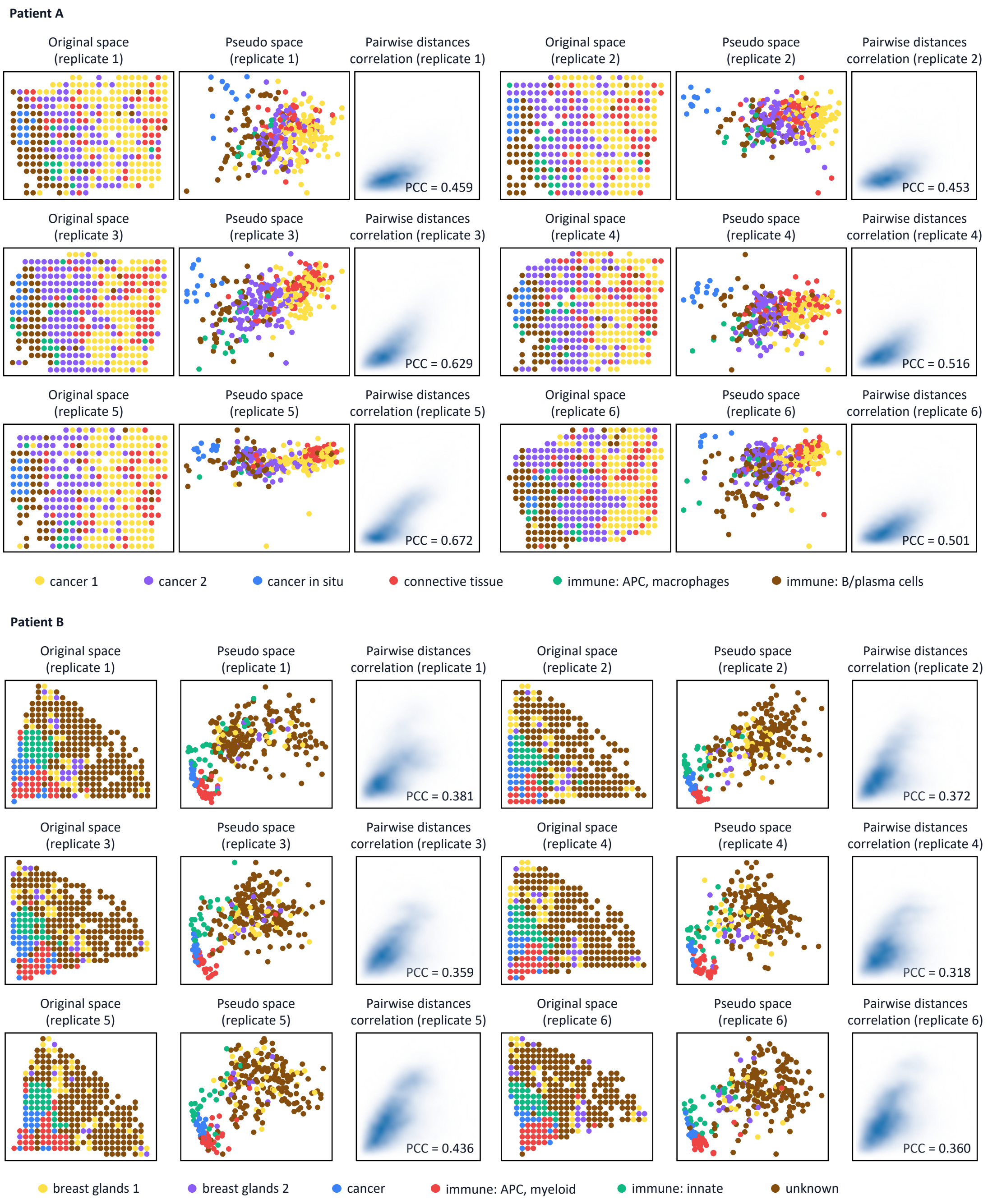

Supplementary Fig. 9. Revealing the shared spatial coherence between replicates of ST data from different patients (A and B) of the human HER2 breast cancer (BC).

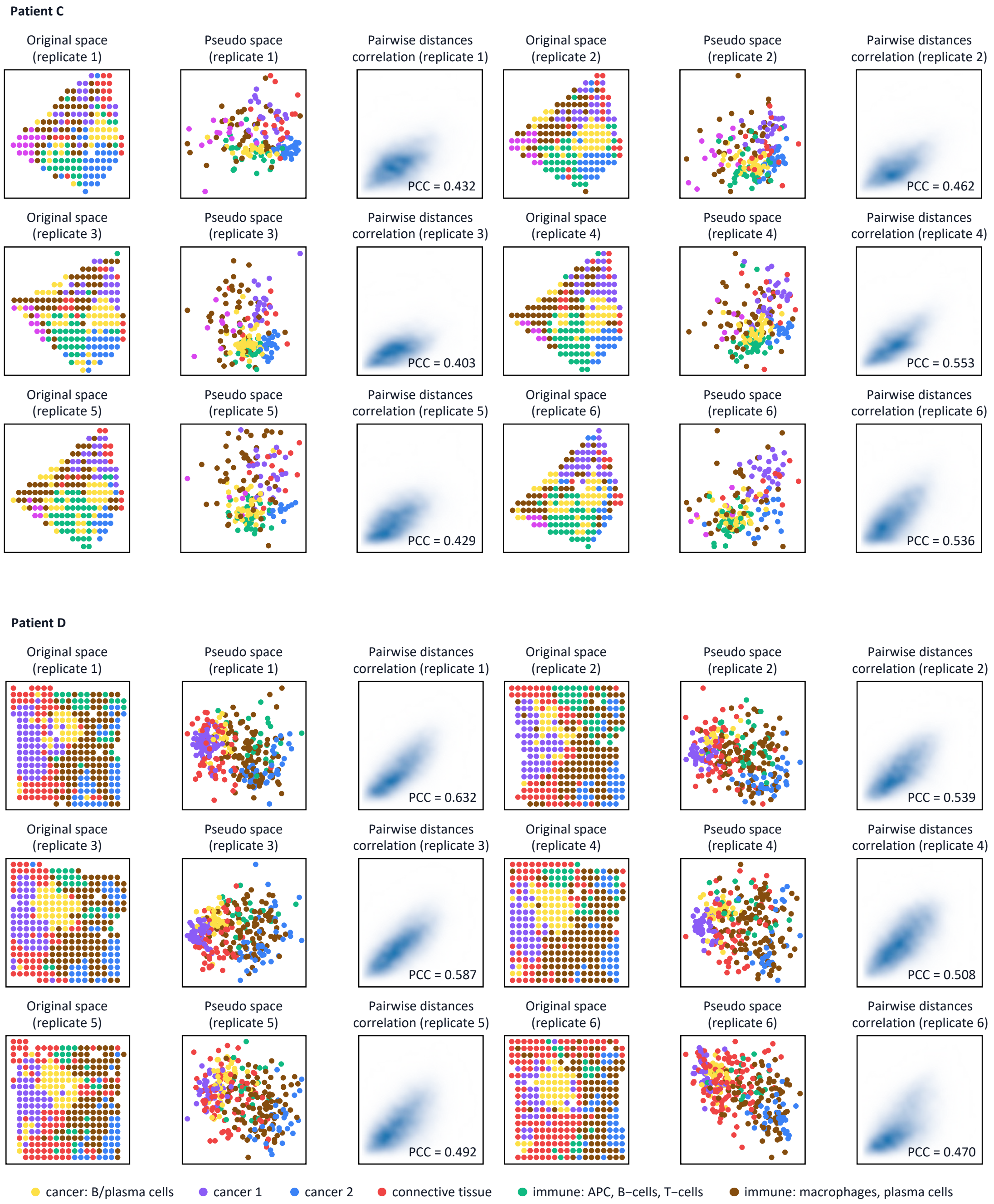

Supplementary Fig. 10. Revealing the shared spatial coherence between replicates of ST data from different patients (C and D) of the human HER2 breast cancer (BC).

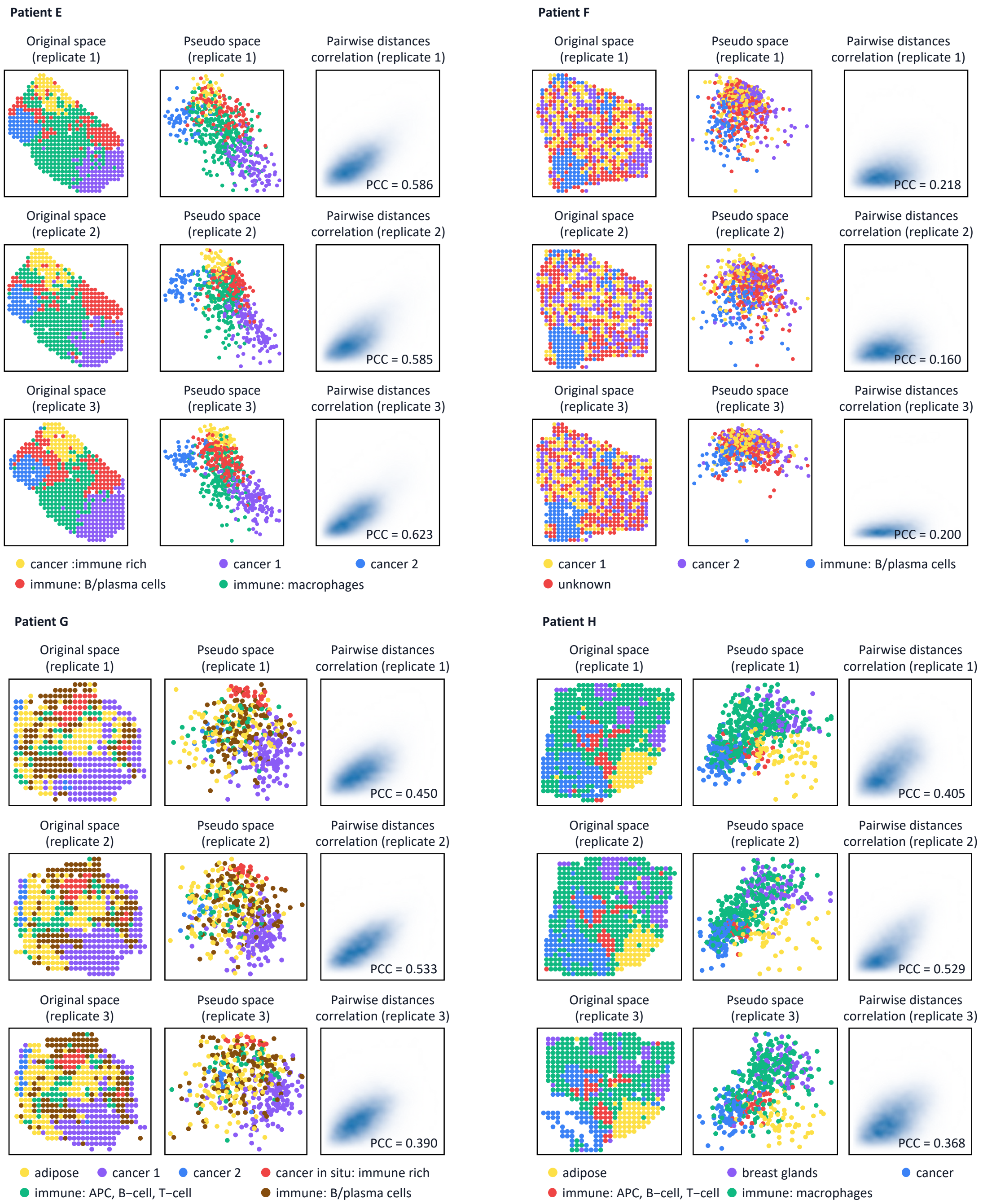

Supplementary Fig. 11. Revealing the shared spatial coherence between replicates of ST data from different patients (E, F, G, and H) of the human HER2 breast cancer (BC).

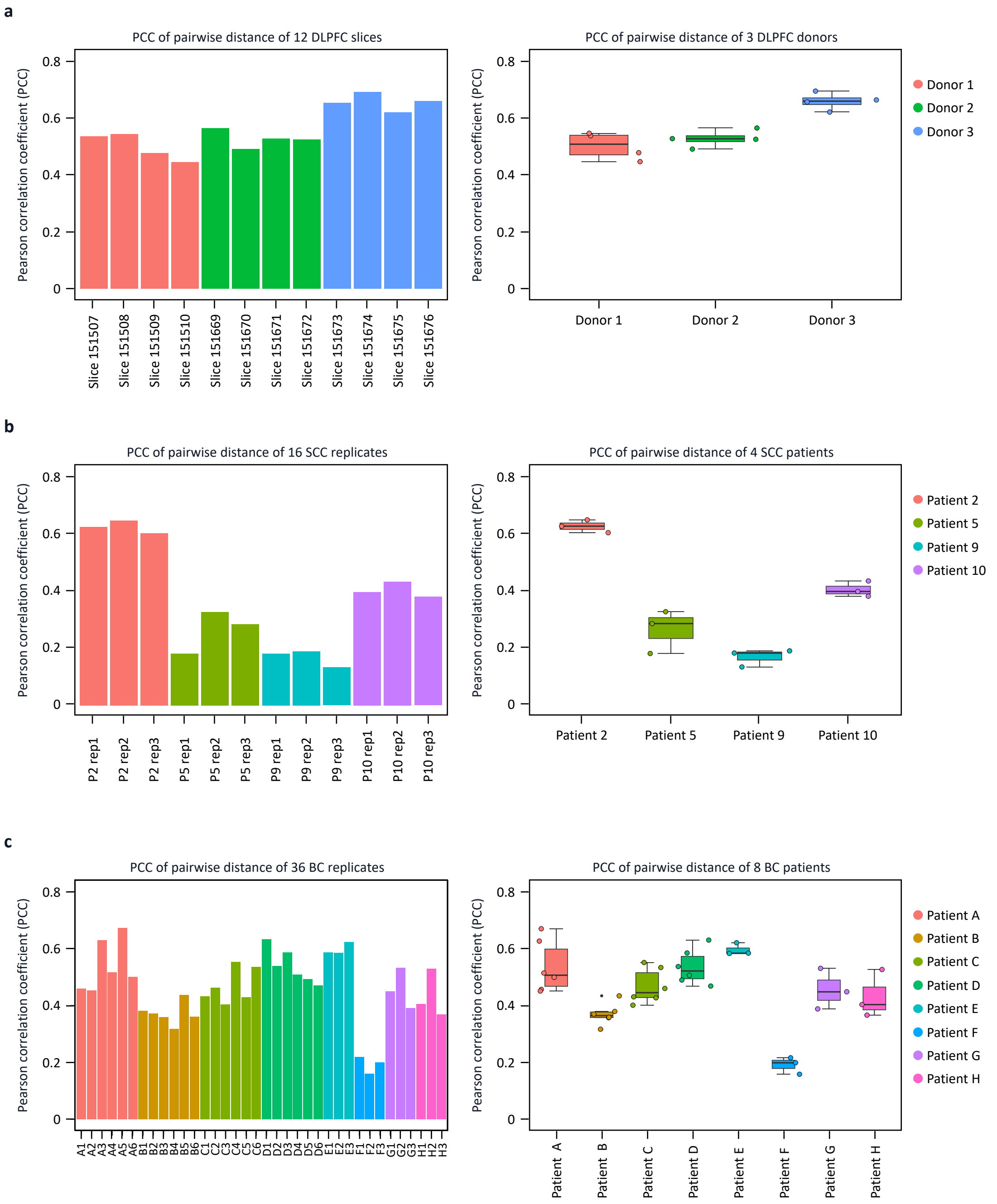

Supplementary Fig. 12. Performance of spatial reconstruction of scSpace on spatial transcriptomics data. The Pearson Correlation Coefficient (PCC) of pairwise distance of spots in original space and pseudo space on human DLPFC (**a**), human SCC (**b**), and human BC (**c**) datasets. Data are presented as the boxplots (minima, 25th percentile, median, 75th percentile, and maxima). In boxplot (**a**), the number of data points for each donor are 4; in boxplot (**b**), the number of data points for each patient are 3; in boxplot (**c**), the number of data points for patient A, B, C, and D is 6, for patient E, F, G, and H is 3.

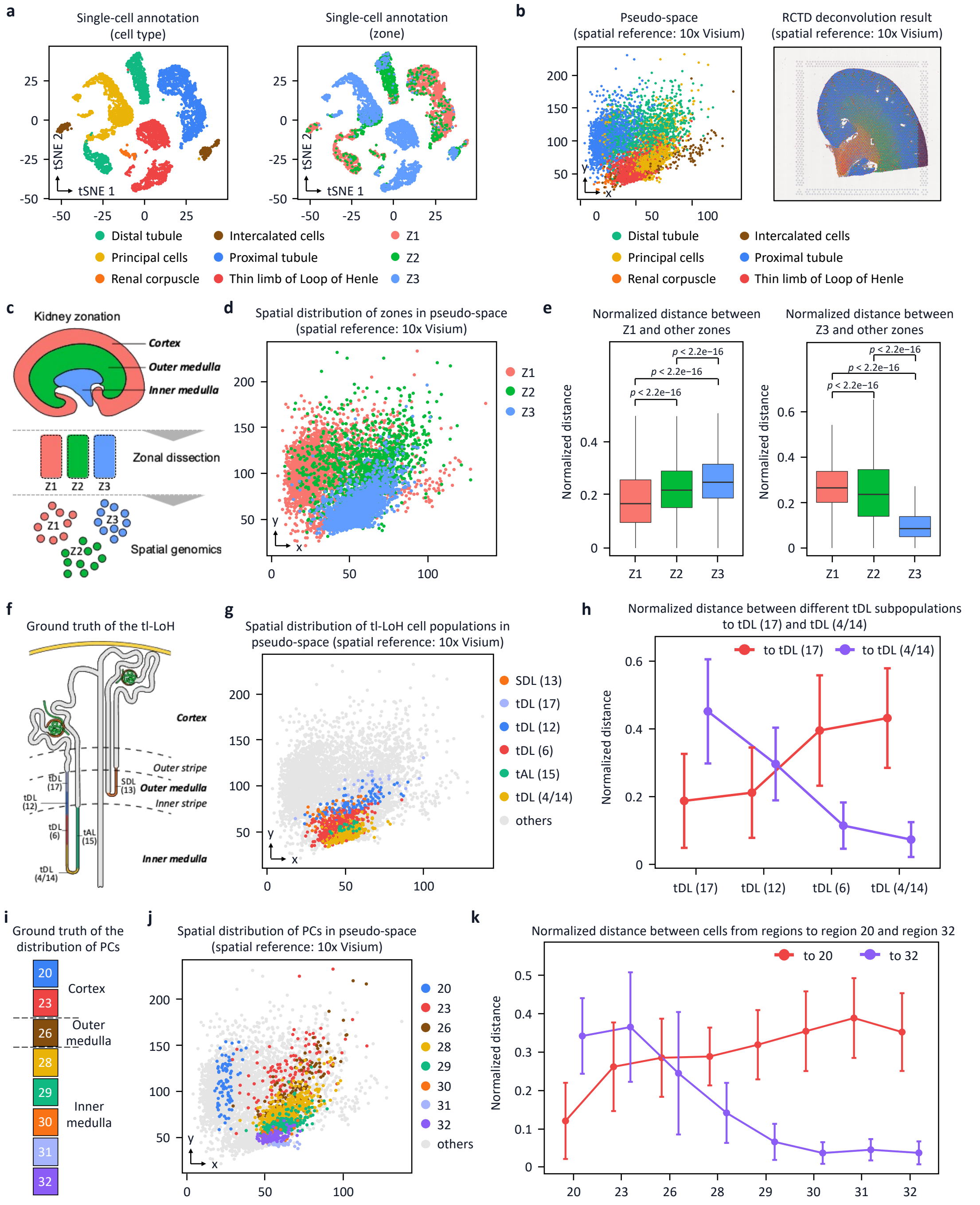

Supplementary Fig. 13. Spatial reconstruction of mouse kidney scRNA-seq data using 10x Visium spatial reference. a, t-SNE visualization of mouse kidney scRNA-seq data, the single-cell annotation was obtained from the original publication, colored by cell types (left) and kidney zones (right). **b,** Comparison of spatial distribution of cell types in the pseudo-space (left) and spatial reference (right). **c,** Schematic of kidney zonation. **d,** Spatial distribution of different kidney zones in the pseudo-space. **e,** Normalized pairwise distance between cells from Z1 (or Z3) and other zones. Data are presented as the boxplots (minima, 25th percentile, median, 75th percentile, and maxima). The number of data points for the normalized pairwise distances to Z1 are 3,288,330, 3,374,224, and 6,820,240, respectively; the number of data points for the normalized pairwise distances to Z3 are 6,820,240, 3,500,560, and 3,539,130, respectively. *P*-value is calculated with the two-sided Wilcoxon rank-sum test (the exact P-value are both 0). **f,** Schematic map indicating anatomic

position for cell populations of thin limb of loop of Henle (tl-LOH). **g,** Spatial distribution of different tl-LOH cell populations in the pseudo-space. All other cell populations are labeled as “others”. **h,** Normalized pairwise distance between different tDL subpopulations to tDL (17) (red) and tDL (4/14) (purple). Data are presented as the median ± SD. The number of data points for the normalized pairwise distances to tDL (17) from left to right are 780, 4,173, 20,241, and 29,445, respectively; the number of data points for the normalized pairwise distances to tDL (4/14) from left to right are 29,445, 80,785, 391,845, and 285,390, respectively. **i,** Schematic map indicating anatomic position and ontology terms for principal cells (PCs) of the ureteric epithelium. **j,** Spatial distribution of different principal cells (PCs) of the ureteric epithelium in the pseudo-space. All other cell populations are labeled as “others”. **k,** Normalized pairwise distance between different PC regions to region 20 (red) and region 32 (purple). Data are presented as the median ± SD. The number of data points for the normalized pairwise distances to 20 from left to right are 3,741, 11,610, 11,352, 43,258, 37,840, 6,020, 7,912, and 8,428, respectively; the number of data points for the normalized pairwise distances to 32 from left to right are 8,428, 13,230, 12,936, 49,294, 43,120, 6,860, 9,016, and 4,851, respectively.

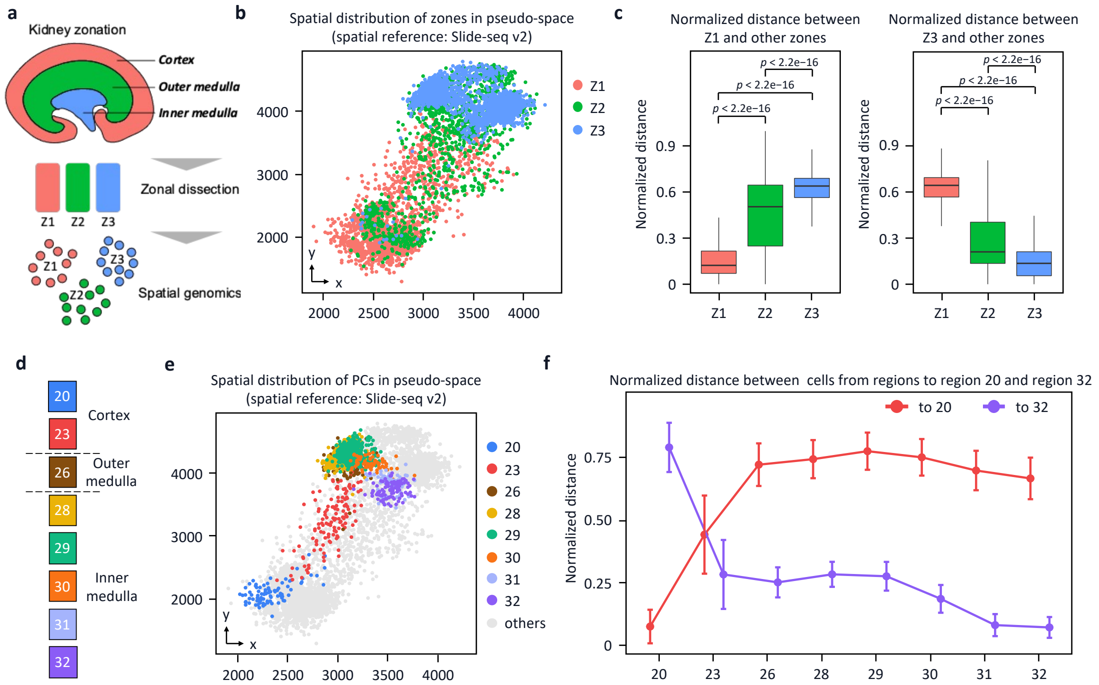

Supplementary Fig. 14. Spatial reconstruction of mouse kidney scRNA-seq data using Slide-seq v2 spatial reference. a, Schematic of kidney zonation. **b,** Spatial distribution of different kidney zones in the pseudo-space. **c,** Normalized pairwise distance between cells from Z1 (or Z3) and other zones. Data are presented as the boxplots (minima, 25th percentile, median, 75th percentile, and maxima). The number of data points for the normalized pairwise distances to Z1 are 3,288,330, 3,374,224, and 6,820,240, respectively; the number of data points for the normalized pairwise distances to Z3 are 6,820,240, 3,500,560, and 3,539,130, respectively. *P*-value is calculated with the two-sided Wilcoxon rank-sum test (the exact P-value are both 0). **d,** Schematic map indicating anatomic position and ontology terms for principal cells (PCs) of the ureteric epithelium. **e,** Spatial distribution of different principal cells (PCs) of the ureteric epithelium in the pseudo-space. All

other cell populations are labeled as “others”. **f,** Normalized pairwise distance between different PC regions to region 20 (red) and region 32 (purple). Data are presented as the median ± SD. The number of data points for the normalized pairwise distances to 20 from left to right are 3,741, 11,610, 11,352, 43,258, 37,840, 6,020, 7,912, and 8,428, respectively; the number of data points for the normalized pairwise distances to 32 from left to right are 8,428, 13,230, 12,936, 49,294, 43,120, 6,860, 9,016, and 4,851, respectively.

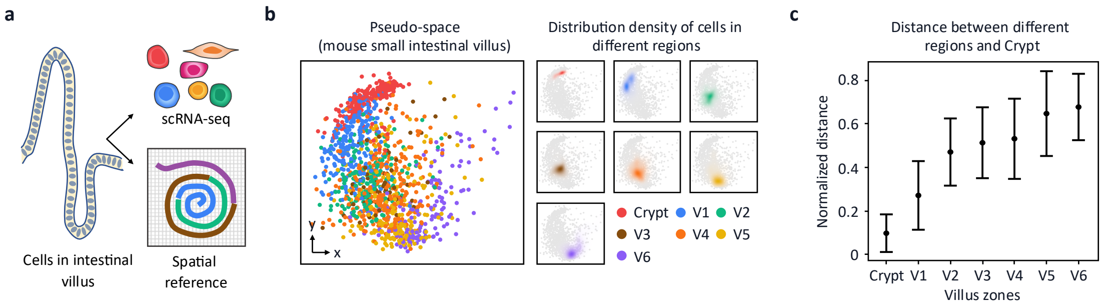

Supplementary Fig. 15. Spatial reconstruction of mouse small intestinal villus scRNA-seq data. a, Schematic of the spatial reconstruction of intestinal epithelium along the Villus Axis. **b,** The pseudo-space of mouse small intestinal villus scRNA-seq data (left) and the spatial distribution of cells in different regions (right). **c,** Normalized pairwise distance between cells from different regions to region Crypt. Data are presented as the median ± SD. The number of data points for the normalized pairwise distances to Crypt from left to right are 27,730, 58,045, 39,480, 15,980, 73,320, 49,115, and 33,840, respectively.

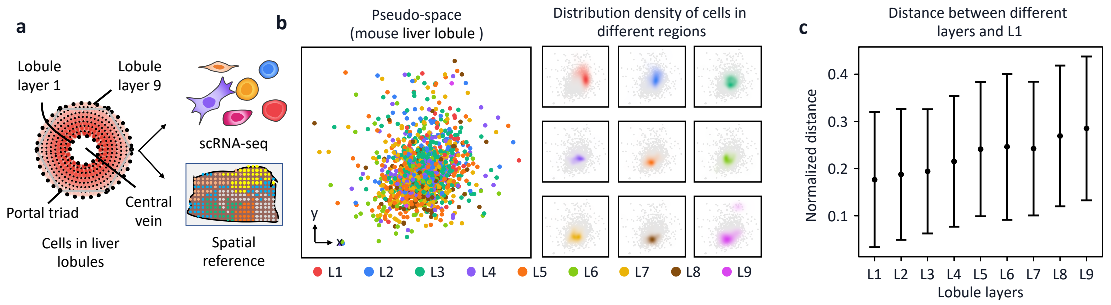

Supplementary Fig. 16. Spatial reconstruction of mouse liver lobule scRNA-seq data. a, Schematic of the spatial reconstruction of liver lobule. **b,** The pseudo-space of mouse liver lobule scRNA-seq data (left) and the spatial distribution of cells in different layers (right). **c,** Normalized pairwise distance between cells from different layers to L1. Data are presented as the median ± SD. The number of data points for the normalized pairwise distances to L1 from left to right are 741, 3,382, 10,526, 6,574, 10,108, 5,776, 11,932, 3,838, and 190, respectively.

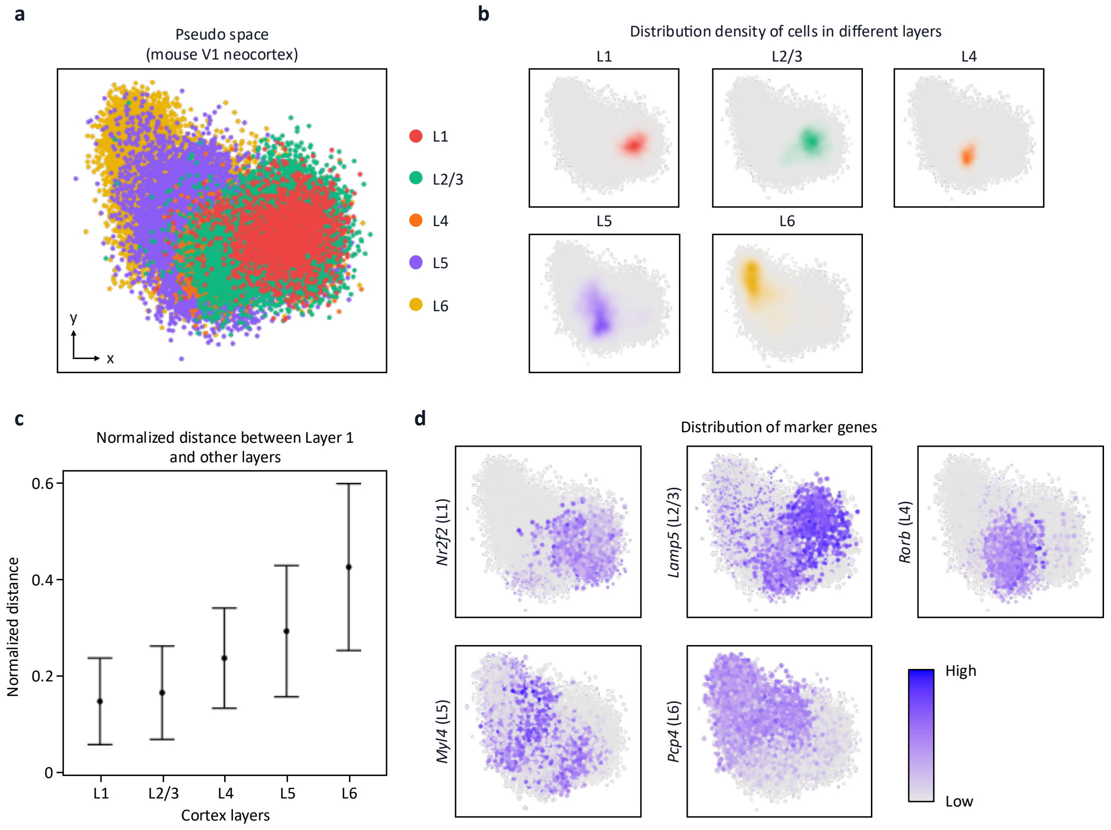

Supplementary Fig. 17. Spatial reconstruction of mouse V1 neocortex scRNA-seq data. a, The pseudo-space of mouse V1 neocortex scRNA-seq data. **b,** The spatial distribution of cells in different layers. **c,** Normalized pairwise distance between cells from different layers to L1. Data are presented as the median ± SD. The number of data points for the normalized pairwise distances to L1 from left to right are 1,682,695, 14,855,400, 4,975,642, 17,213,924, and 18,708,634, respectively. **d,** The spatial expression patterns of marker genes of each layer in the pseudo-space.

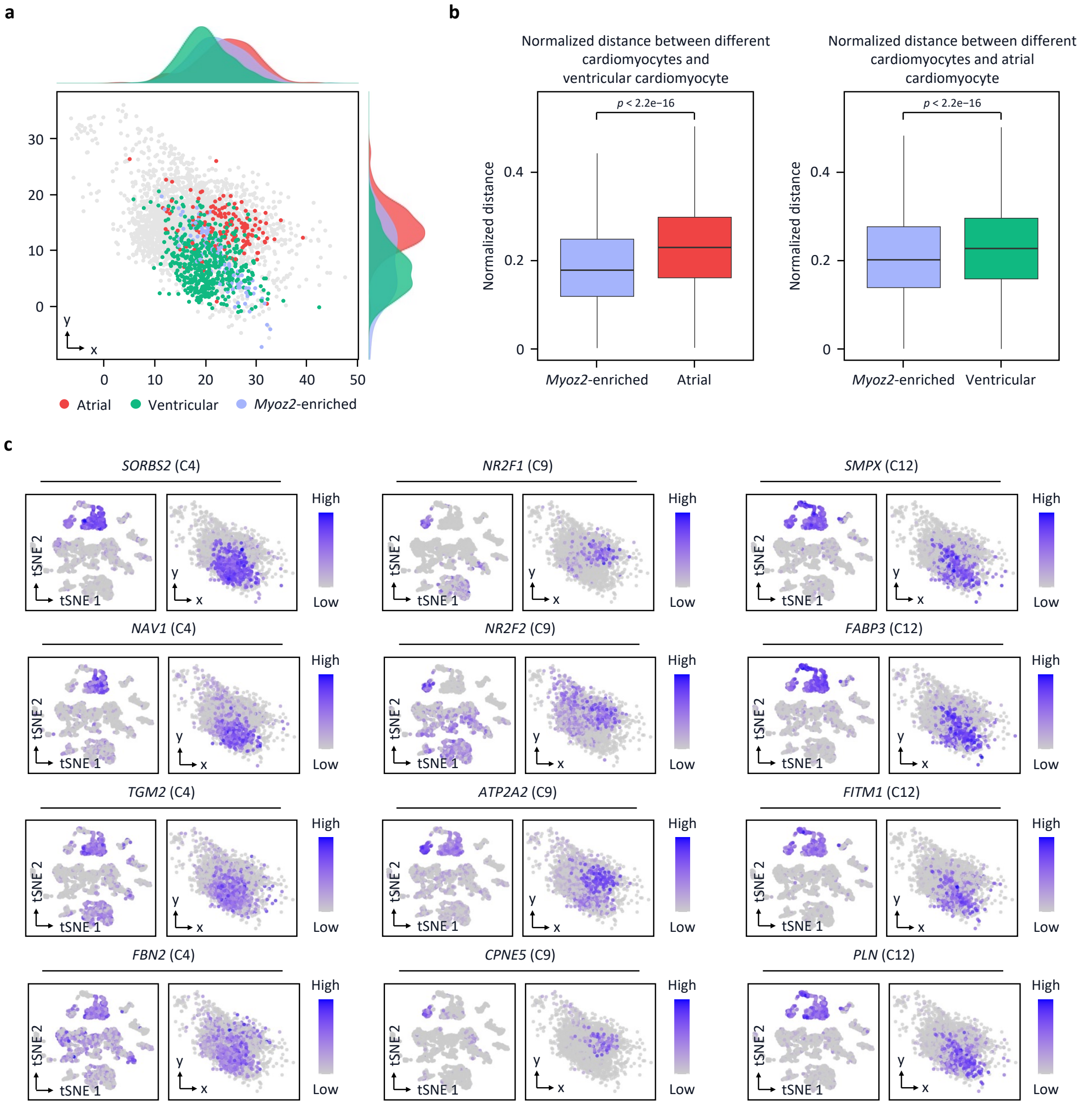

Supplementary Fig. 18. Spatial reconstruction of human embryonic heart scRNA-seq data. a, Spatial distribution of different cardiomyocyte subpopulations in the pseudo-space. All other cell populations are colored with grey. **b,** Normalized pairwise distance between different cardiomyocyte subpopulations to ventricular cardiomyocyte (left) and atrial cardiomyocyte (right). Data are presented as the boxplots (minima, 25th percentile, median, 75th percentile, and maxima). The number of data points for the normalized pairwise distances to ventricular cardiomyocyte are 48,209 and 75,544, respectively; the number of data points for the normalized pairwise distances to atrial cardiomyocyte are 14,744 and 75,544, respectively. *P*-value is calculated with the two-sided Wilcoxon rank-sum test (the exact P-values from left to right are 0 and 7.0e-93, respectively). **c,** Expression patterns of marker genes of different cardiomyocyte subpopulations in the t-SNE (left) and pseudo-space (right).

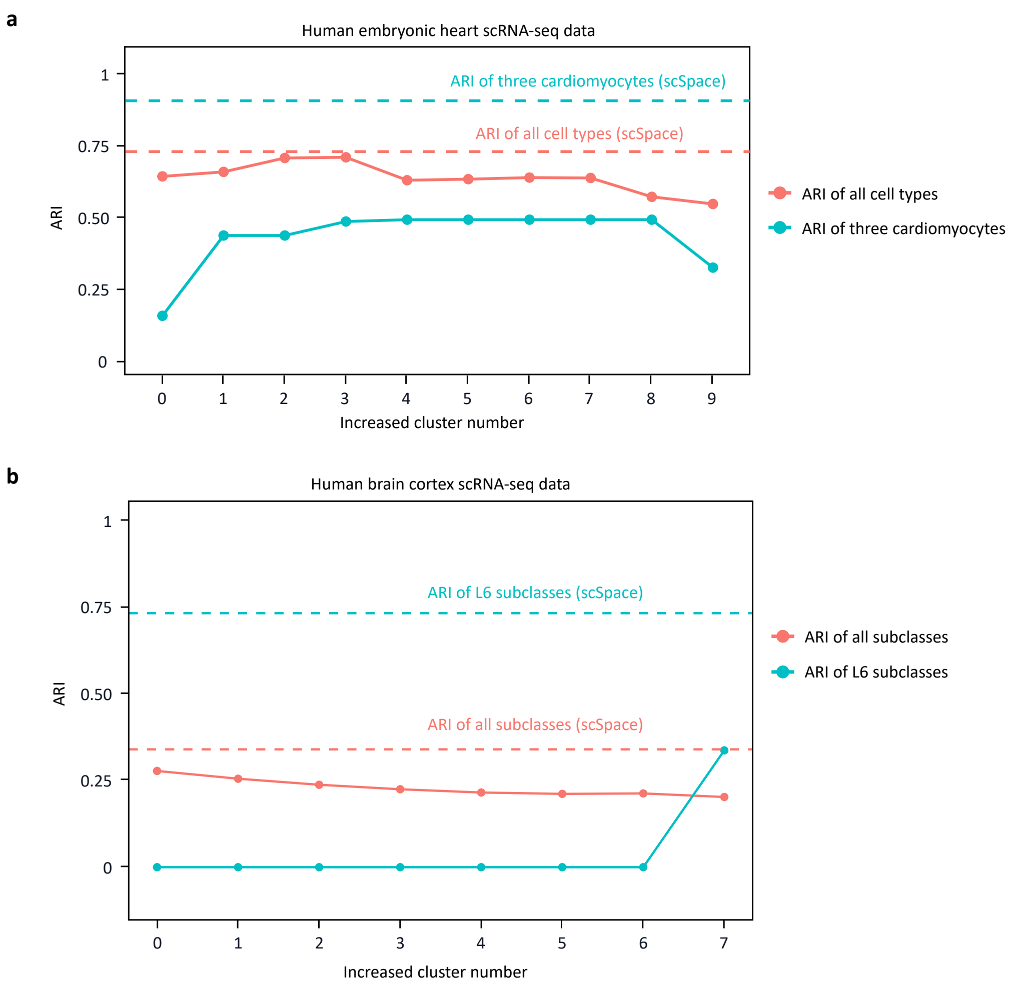

Supplementary Fig. 19. Exploration of the influence of targeted clustering number on the clustering accuracy of Seurat. a, Clustering accuracy of Seurat in all cell types (red) and cardiomyocyte subpopulations (blue) under different targeted clustering number on human embryonic heart scRNA-seq data. **b,** Clustering accuracy of Seurat in all cell types (red) and L6 subclasses (blue) under different targeted clustering number on human brain cortex scRNA-seq data.

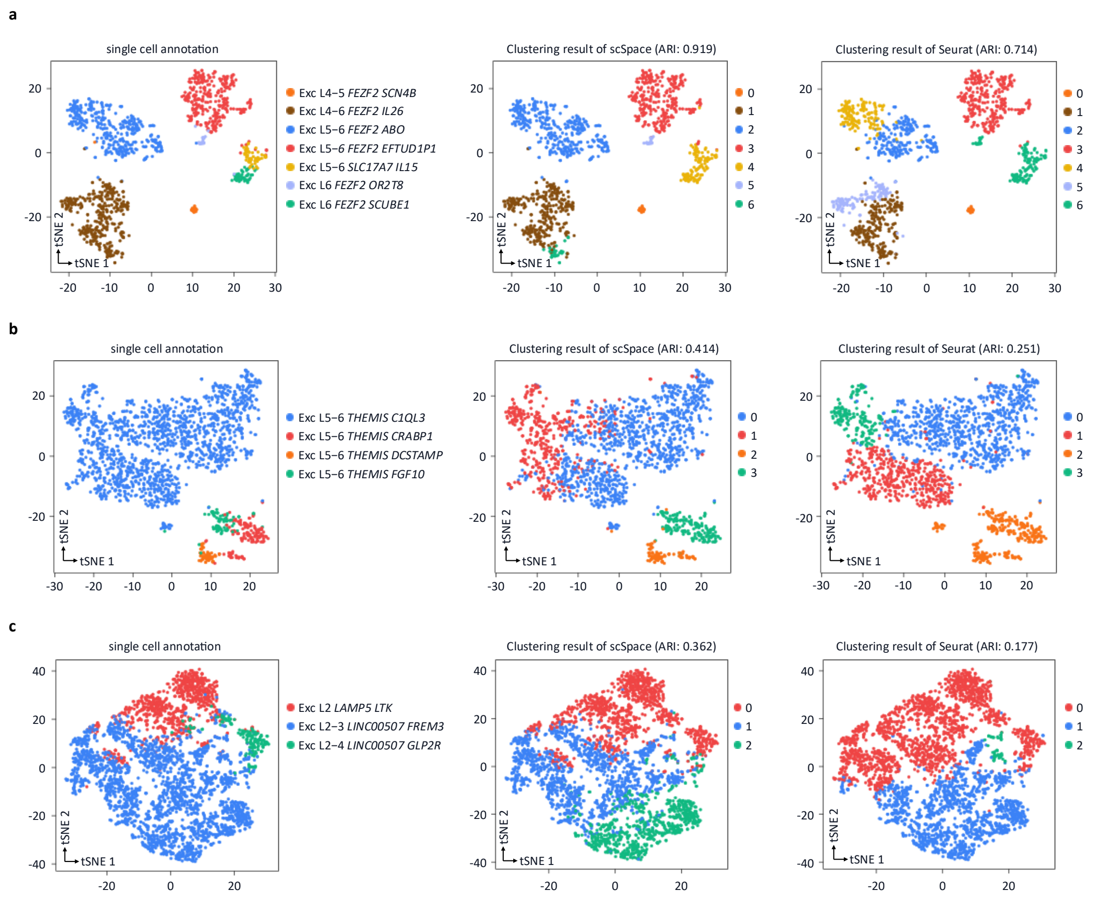

Supplementary Fig. 20. Comparison of clustering accuracy between scSpace and Seurat on other three types of excitatory neuron subclasses. Comparison of clustering accuracy between scSpace and Seurat on *FEZF2*-expressing (**a**), *THEMIS*-expressing (**b**), and *LAMP5*-expressing (**c**) excitatory neuron subclasses.

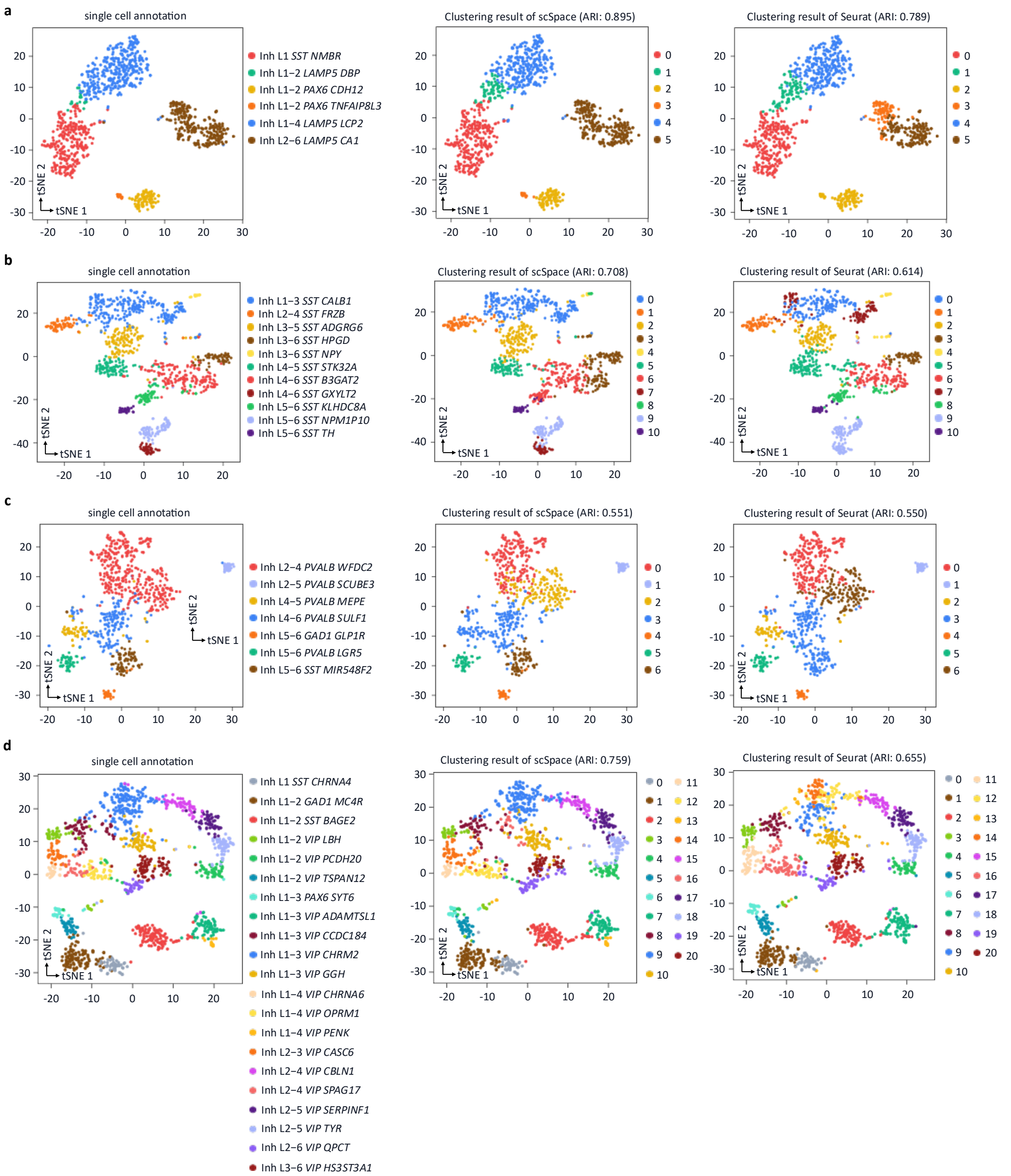

Supplementary Fig. 21. Comparison of clustering accuracy between scSpace and Seurat on other four types of inhibitory neuron subclasses. Comparison of clustering accuracy between scSpace and Seurat on *LAMP5 PAX6* (**a**), *SST* (**b**), *PVALB* (**c**), and *VIP* (**d**) inhibitory neuron subclasses.

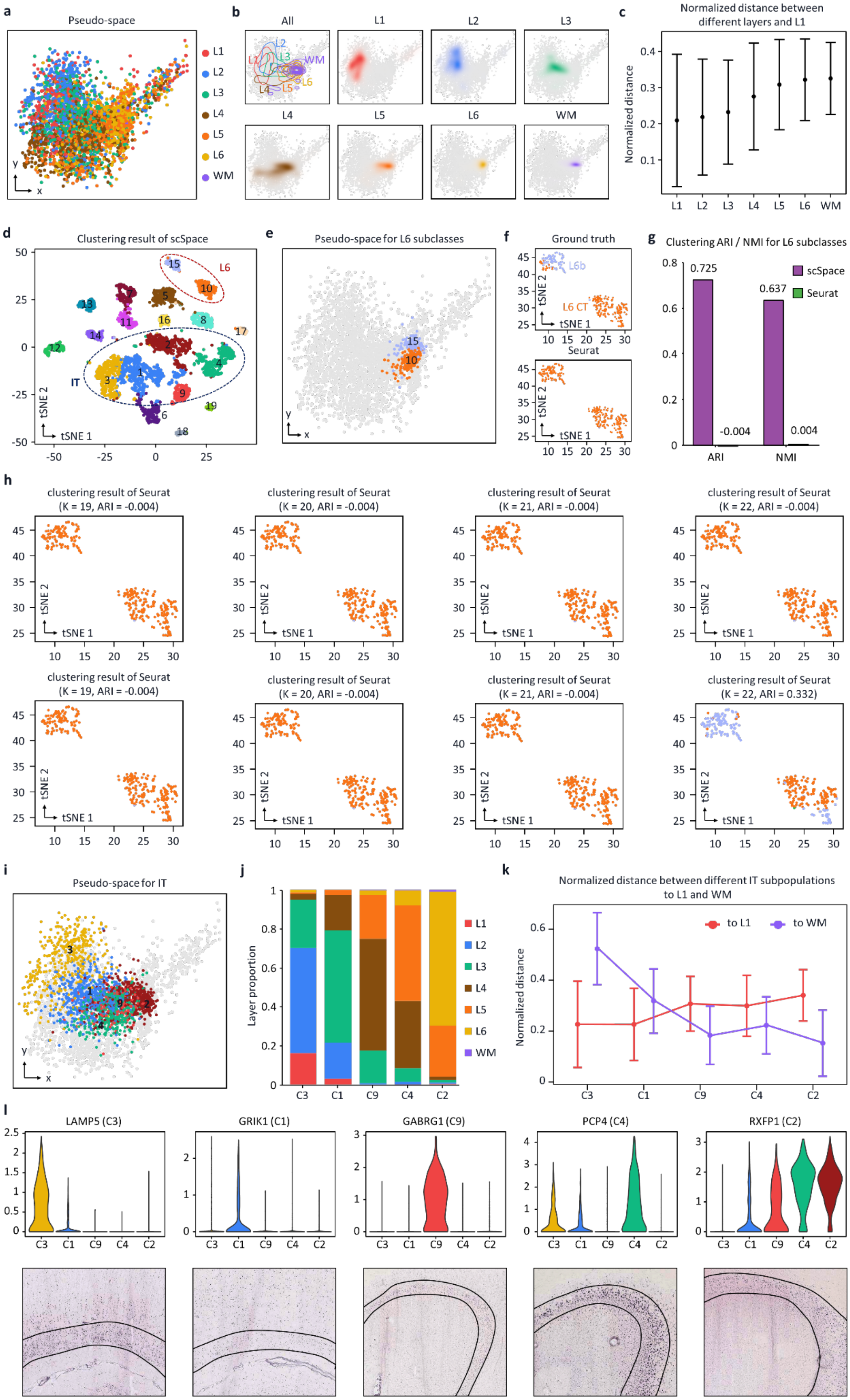

a
Pseudo-space
L1
L2
L3
L4
L5
L6
WM
b
All
L1
L2
L3
L4
L5
L6
WM
c
Normalized distance between different layers and L1
Normalized distance
d
Clustering result of scSpace
L6
IT
tSNE 1
tSNE 2
e
Pseudo-space for L6 subclasses
f
Ground truth
L6b
L6 CT
Seurat
g
Clustering ARI / NMI for L6 subclasses
0.725
0.637
-0.004
0.004
scSpace
Seurat
ARI
NMI
h
clustering result of Seurat (K = 19, ARI = -0.004)
clustering result of Seurat (K = 20, ARI = -0.004)
clustering result of Seurat (K = 21, ARI = -0.004)
clustering result of Seurat (K = 22, ARI = -0.004)
clustering result of Seurat (K = 19, ARI = -0.004)
clustering result of Seurat (K = 20, ARI = -0.004)
clustering result of Seurat (K = 21, ARI = -0.004)
clustering result of Seurat (K = 22, ARI = 0.332)
i
Pseudo-space for IT
j
Layer proportion
C3
C1
C9
C4
C2
k
Normalized distance between different IT subpopulations to L1 and WM
to L1
to WM
Normalized distance
l
LAMP5 (C3)
GRIK1 (C1)
GABRG1 (C9)
PCP4 (C4)
RXFP1 (C2)

Supplementary Fig. 22. Discovery of fine subpopulations in human cortex by scSpace. a, The pseudo-space of human cortex scRNA-seq data. **b,** The spatial distribution of cells in different layers in the pseudo-space. **c,** Normalized pairwise distance between cells from different layers to L1. Data are presented as the median ± SD. The number of data points for the error bars from left to right are 47,586, 168,476, 231,000, 226,996, 234,696, 267,960, and 8,008, respectively. **d,** t-SNE visualization of human cortex scRNA-seq data, colored by the clusters identified by scSpace. **e,** Spatial distribution of two subclasses of L6 (L6b and L6 CT) in the pseudo-space. All other subclasses are labeled as "others". **f,** t-SNE visualization of two L6 subclasses with the original annotation (top) and scSpace's clustering result (bottom). **g,** Performance comparison of clustering result for two L6 subclasses between scSpace and Seurat. **h,** t-SNE visualization of two L6 subclasses with Seurat's clustering result under different targeted clustering number setting. **i,** The spatial distribution of different IT subpopulations identified by scSpace in the pseudo-space. All other cell types are colored with grey. **j,** The distribution proportion of each IT subpopulation on each cortex layer. **k,** Normalized pairwise distance between different IT subpopulations to L1 (red) and WM (purple). Data are presented as the median ± SD. The number of data points for the normalized pairwise distances to L1 from left to right are 121,968, 174,020, 58,212, 121,600, and 139,832, respectively; the number of data points for the normalized pairwise distances to WM from left to right are 10,296, 14,690, 4,914, 10,270, and 11,804, respectively. **l,** Expression value of marker genes of different IT subpopulations (top) and validation of their spatial expression patterns on ISH data downloaded from Allen Brain Atlas (bottom).

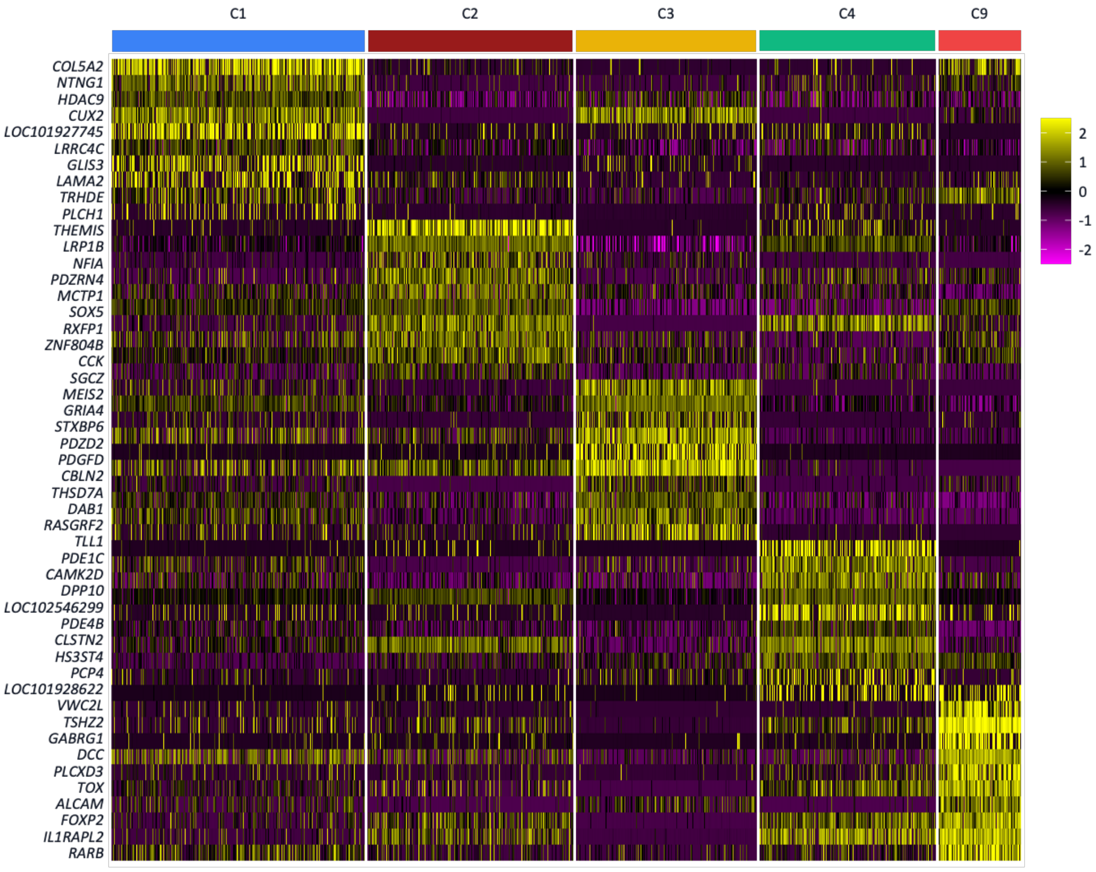

Supplementary Fig. 23. Heatmap of top 10 differentially expressed genes of each IT subpopulations.

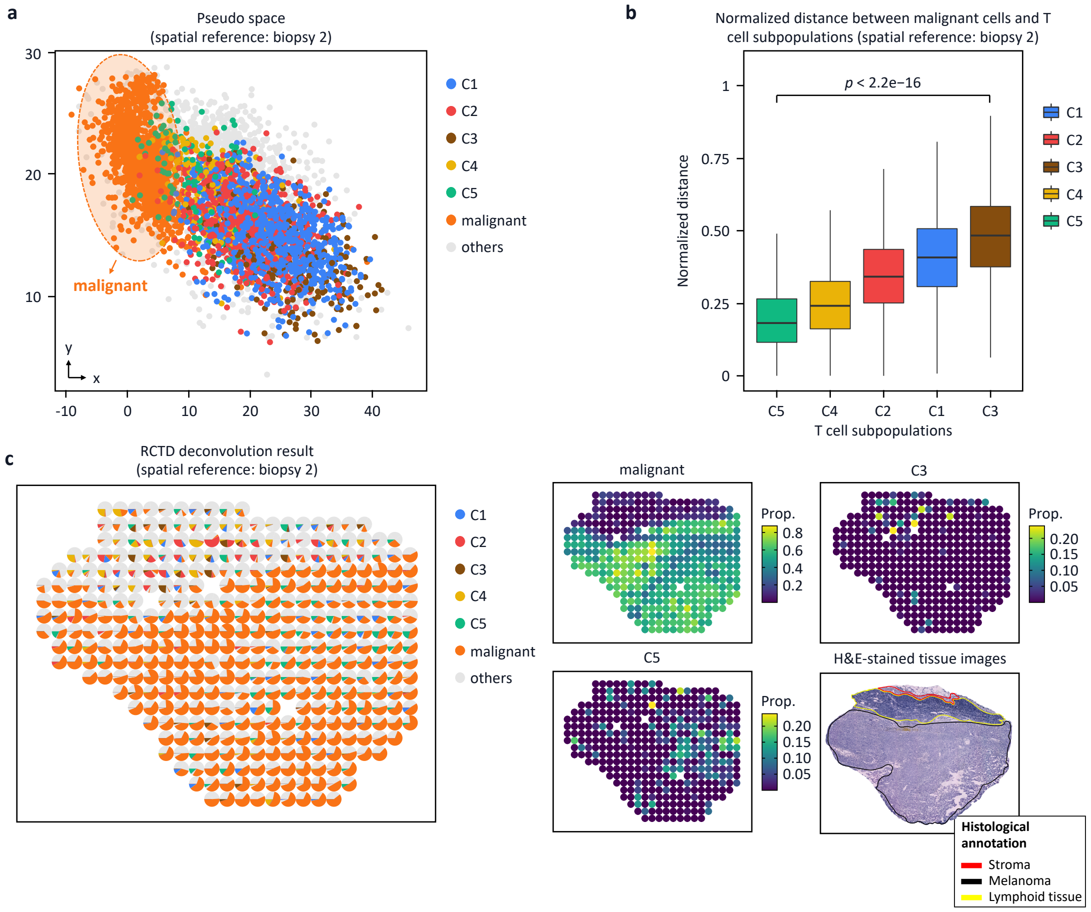

Supplementary Fig. 24. Spatial reconstruction of T cell subpopulations in melanoma using another spatial reference. a, The spatial distribution of T cell subpopulations and malignant cells in the pseudo-space using the spatial transcriptomics data from biopsy 2 as the spatial reference. All other cell types are labeled as “others”. **b,** Normalized pairwise distance between different T cell subpopulations to malignant cells. Data are presented as the boxplots (minima, 25th percentile, median, 75th percentile, and maxima). The number of data points for the normalized pairwise distances to malignant cells are 144,555, 251,400, 731,574, 1,148,898 and 318,021, respectively. *P*-value is calculated with the two-sided Wilcoxon rank-sum test (the exact P-value is 0). **c,** The spatial distribution of C3 and C5 subpopulations in the spatial transcriptomics reference from biopsy 2 calculated by RCTD. All other cell types are labeled as “others”.

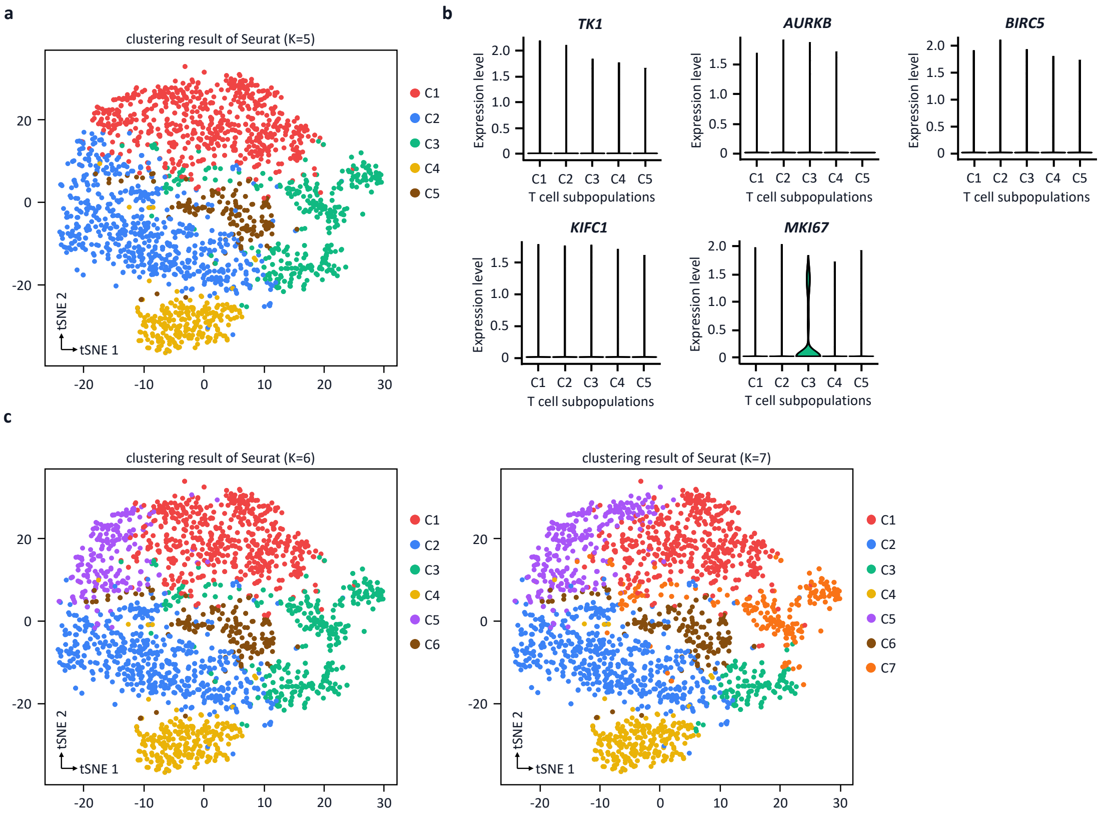

Supplementary Fig. 25. Subcluster results of T cells by Seurat. a, t-SNE visualization of five T cell subpopulations identified by Seurat. **b,** Expression level of melanoma-related genes in each T cell subpopulation identified by Seurat. **c,** t-SNE visualization of six (left) and seven (right) T cell subpopulations identified by Seurat.

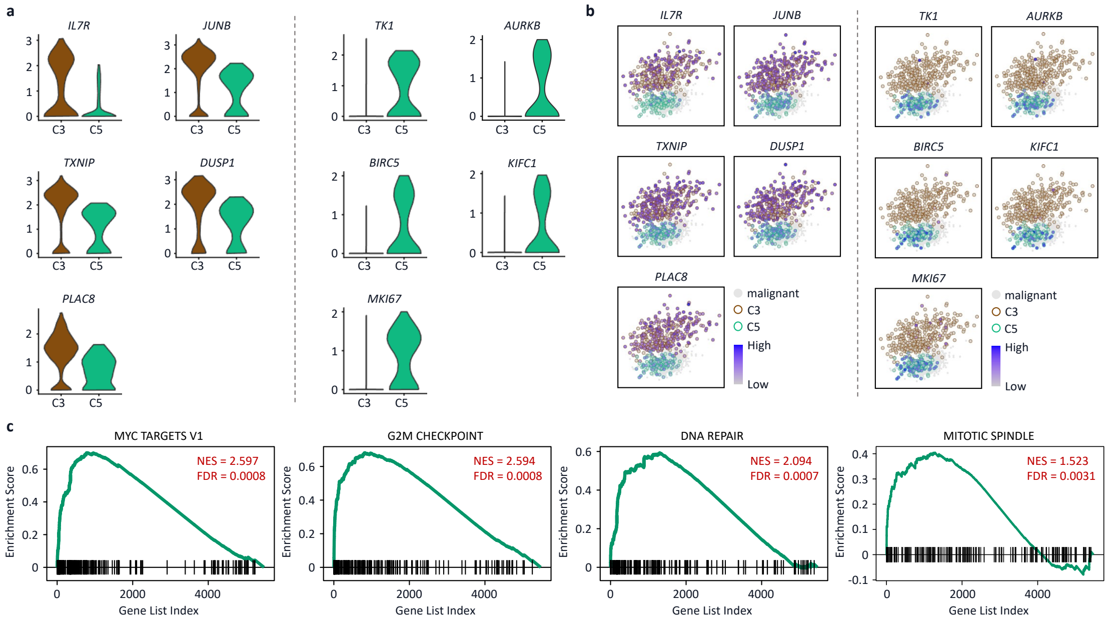

Supplementary Fig. 26. Spatial analysis T cell subpopulations in melanoma. a, Vlnplot of marker genes of C3 and C5 T cell subpopulations. **b,** Feature plot of marker genes of C3 and C5 T cell subpopulations. **c,** Gene set enrichment analysis (GSEA) result of differentially expressed genes of C5 T cell subpopulation.

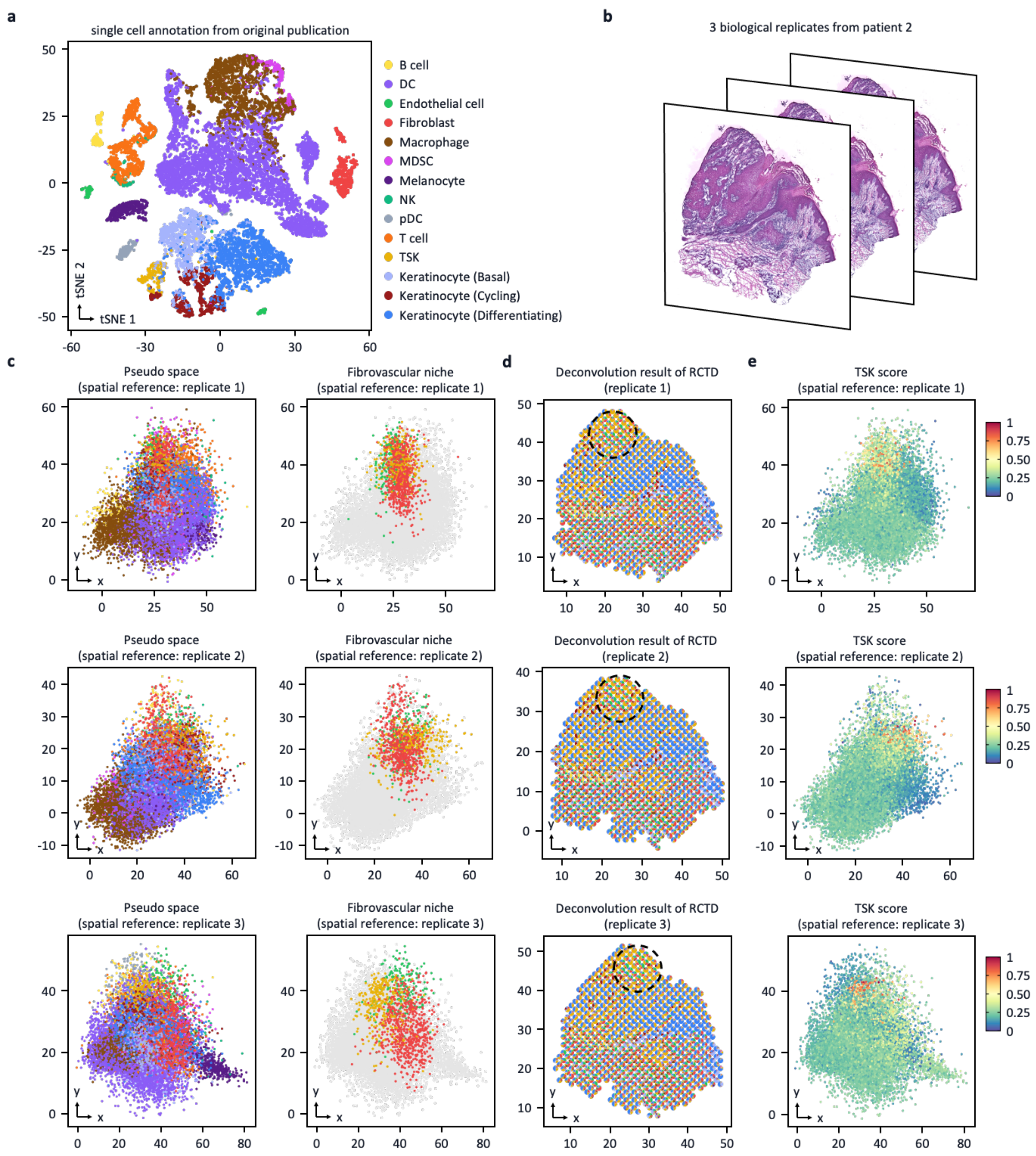

Supplementary Fig. 27. Spatial reconstruction of human SCC scRNA-seq data using scSpace. a, t-SNE visualization of human SCC scRNA-seq data, the single-cell annotation was obtained from the original publication. **b,** The multiple spatial transcriptomics data from SCC patient 2 utilized as the spatial reference. **c,** The reconstructed pseudo-space (left) and the fibrovascular niche (right) of scRNA-seq data by scSpace. **d,** The RCTD deconvolution results of three replicated spatial transcriptomics reference from SCC patient 2. **e,** Expression patterns of TSK score in the pseudo-space.

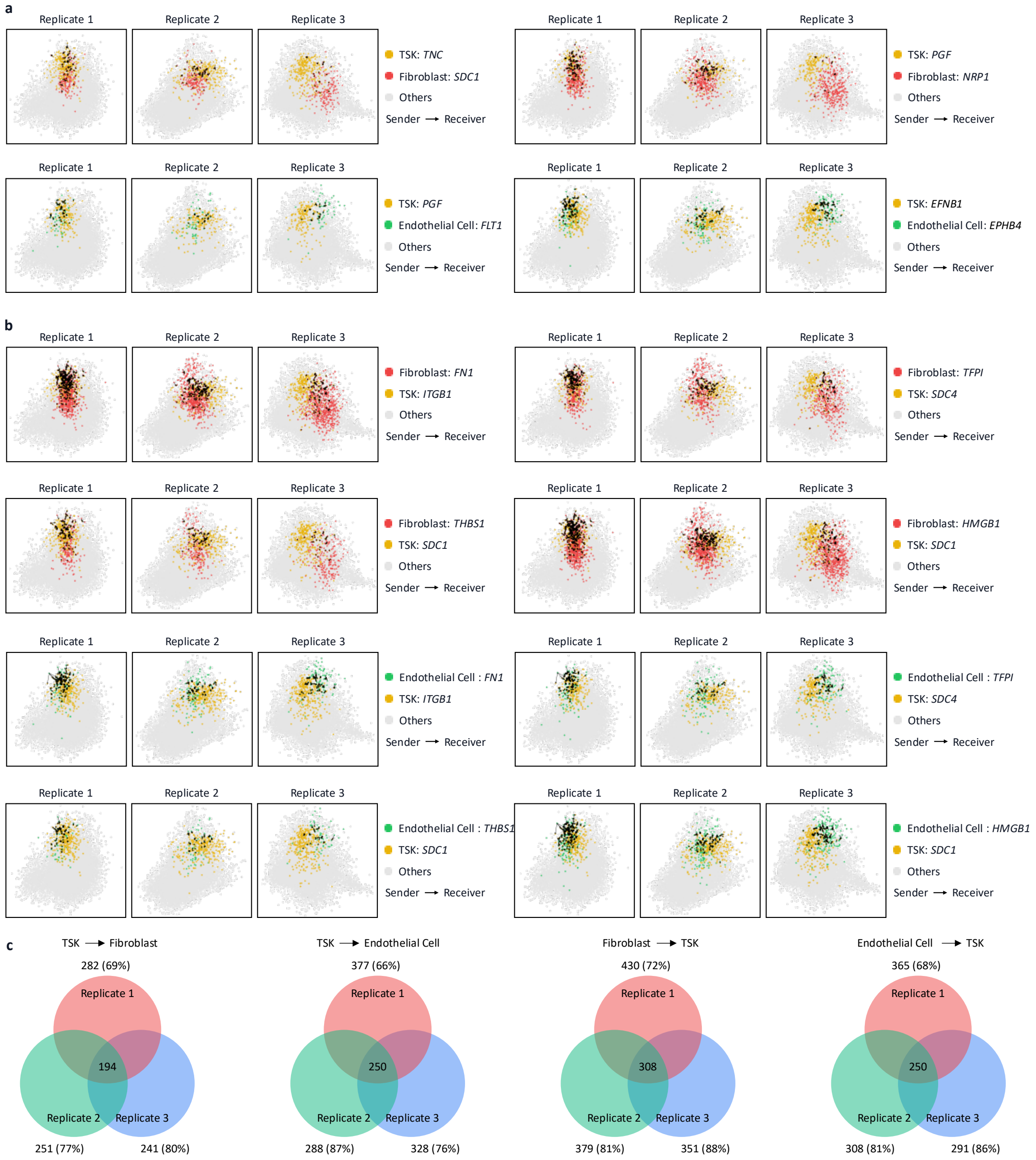

Supplementary Fig. 28. Reconstruction of cellular crosstalk between TSKs and TME cells in pseudo-space. a, Spatial distribution of the inferred LRIs by SpaTalk between the TSK senders to the Fibroblast and Endothelial Cell receivers in the pseudo-space. **b,** Spatial distribution of the inferred LRIs by SpaTalk between the Fibroblast and Endothelial Cell senders to the TSK receivers in the pseudo-space. **c,** Comparison of inferred LRI pairs over three pseudo-spaces constructed by scSpace using three replicates from SCC patient 2.

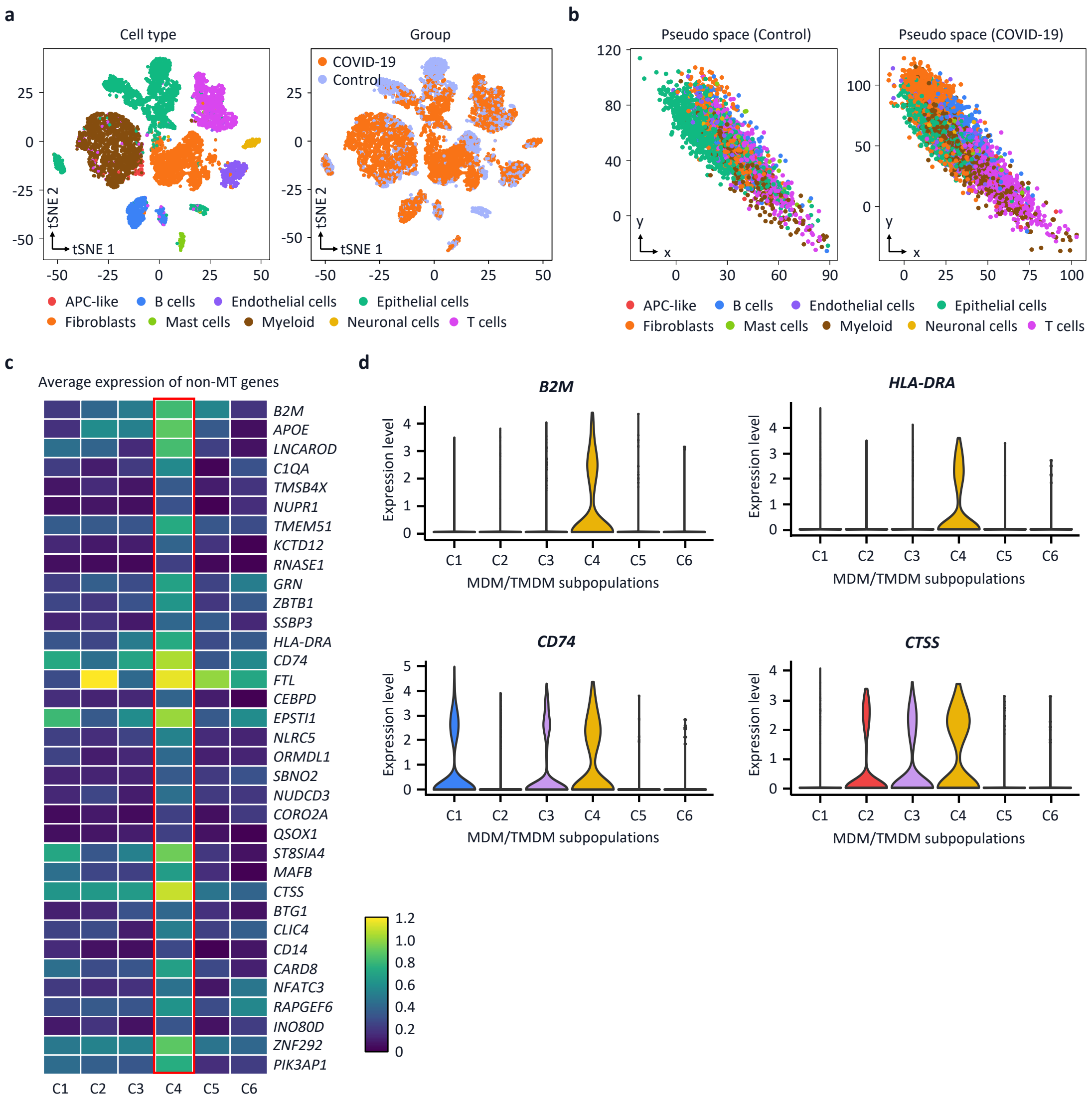

Supplementary Fig. 29. Spatial analysis of the invasion of myeloid subpopulations in COVID-19. **a,** t-SNE visualization of human COVID-19 scRNA-seq data, the single-cell annotation was obtained from the original publication, colored by cell types (left) and group (right). **b,** The reconstructed pseudo-space for Control (left) and COVID-19 (right) group by scSpace. **c,** Averaged expression level of 35 non-mitochondrial genes among differentially expressed genes of C4 in MDM/TMDM subpopulations. **d,** The expression level of four MHC-related genes in MDM/TMDM subpopulations.

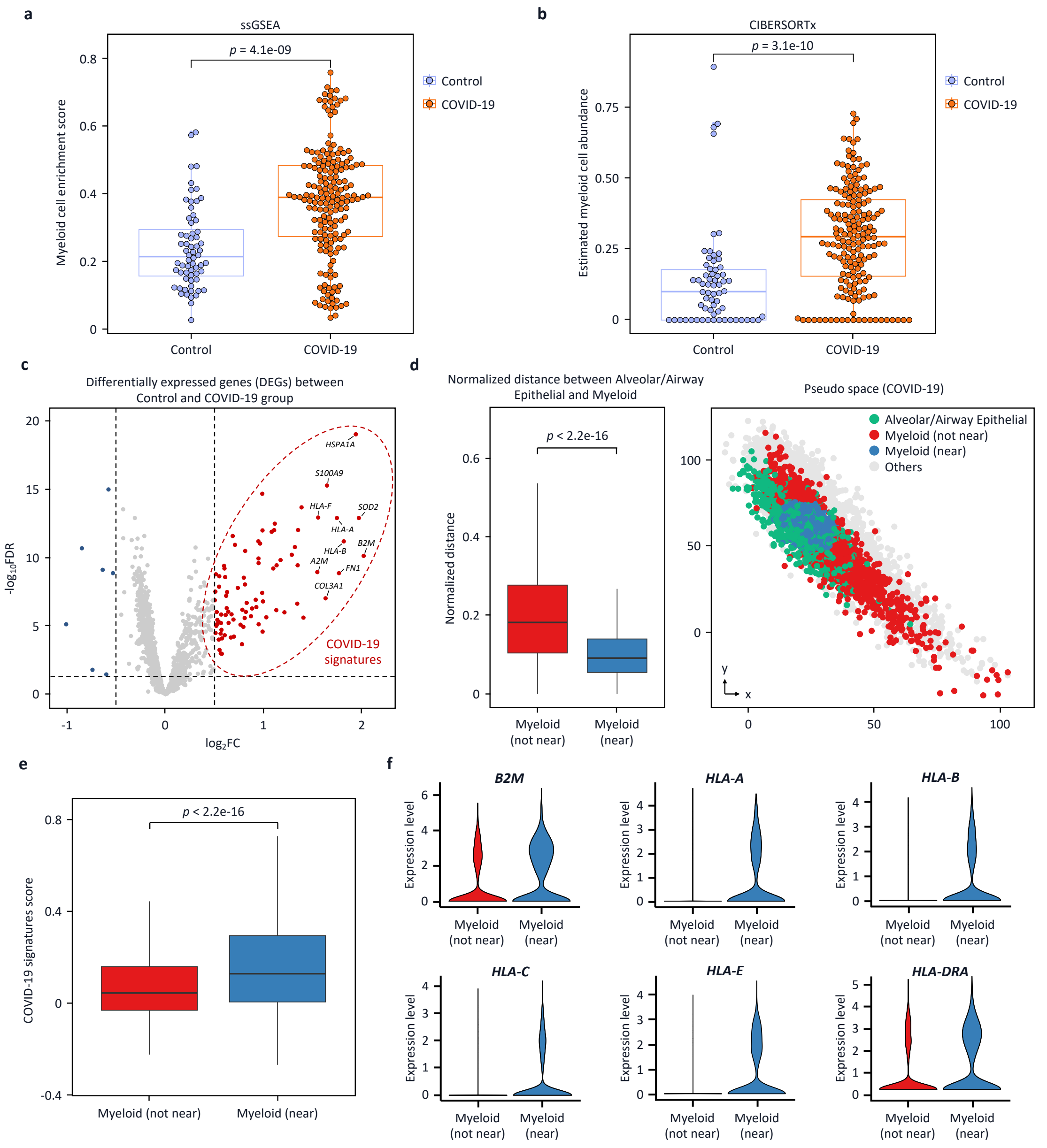

Supplementary Fig. 30. Validation of the spatial variability identified by scSpace on the GeoMx DSP targeted spatial transcriptomics data. a, b, Enrichment score (**a**) and estimated the abundance calculated by the CYBERSORT program (**b**) of myeloid cells for each ROI in the Control (n = 64) and COVID-19 (n = 180) group. Data are presented as the boxplots (minima, 25th percentile, median, 75th percentile, and maxima). *P*-value is calculated with the two-sided Wilcoxon rank-sum test. **c,** Volcano plot of differential gene expressions in COVID-19 group versus Control group. Red and blue points mark the genes with significantly increased or decreased expressions in COVID-19 group (FDR < 0.05 and log2FC > 0.5). **d,** The reconstructed pseudo-space for COVID-19 group by scSpace, wherein myeloid cells were divided into "near to epithelial" and "not near to epithelial" myeloid cells according to the average distance to epithelial cells. All other cell types are labeled as "others". Data are presented as the boxplots (minima, 25th percentile, median, 75th percentile, and maxima). The number of data points for each type of myeloid cells is 1401. *P*-value is calculated with the two-sided Wilcoxon rank-sum test (the exact *P*-value is 0). **e,** Boxplot comparing the COVID-19 signatures scores between two types of myeloid cells. Data are presented as the boxplots

(minima, 25th percentile, median, 75th percentile, and maxima). The number of data points for each type of myeloid cells is 1401. *P*-value is calculated with the two-sided Wilcoxon rank-sum test (the exact *P*-value is 4.1e-20). **f,** Violin plots of expression levels of MHC-related genes in two types of myeloid cells.

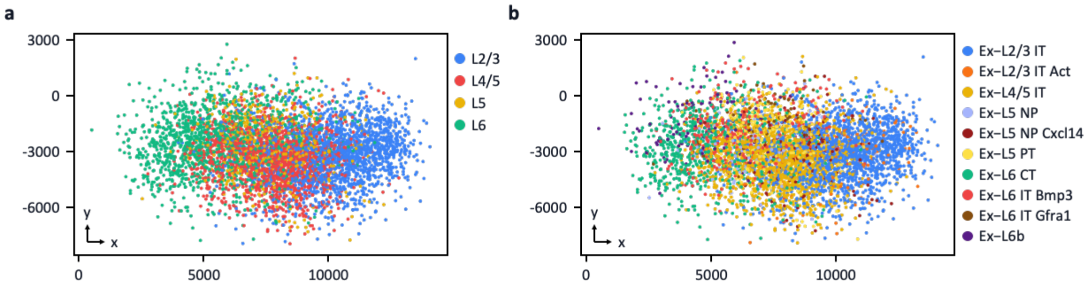

Supplementary Fig. 31. Spatial reconstruction of mouse cerebral cortex scATAC-seq data using scSpace. a, b, The reconstructed pseudo-space of mouse cerebral cortex scATAC-seq data, the single-cell annotation was obtained from the original publication, colored by layer (**a**) and cell types (**b**).
