# Supplementary material for: Reconstruction of the cell pseudo-space from single-cell RNA sequencing data with scSpace
Source: Nat Commun. 2023 Apr 29;14:2484. doi: 10.1038/s41467-023-38121-4 (PMC10148590; doi:10.1038/s41467-023-38121-4)
Supplement: Supplementary file 3 — Description of additional supplementary files [file 41467_2023_38121_MOESM3_ESM.pdf]

Description of additional supplementary files

Supplementary Data 1. Detailed information of datasets used in scSpace.

Supplementary Data 2. Detailed information of the simulated data for benchmarking test.

Supplementary Data 3. The Pearson correlation coefficient of pairwise distances between cells in the pseudo-space and original space over 50 simulated data.

Supplementary Data 4. The differentially expressed genes of five IT neuron subpopulations identified by scSpace (log2FC > 0.5, adjust p < 0.05).

Supplementary Data 5. The complete lists of the ligand-receptorinteraction pairs inferred by SpaTalk which are conserved in three different pseudo-spaces.

Supplementary Data 6. The complete lists of the COVID-19 signatures calculated by limma.
