# Supplementary material for: Reconstruction of the cell pseudo-space from single-cell RNA sequencing data with scSpace
Source: Nat Commun. 2023 Apr 29;14:2484. doi: 10.1038/s41467-023-38121-4 (PMC10148590; doi:10.1038/s41467-023-38121-4)
Supplement: Supplementary file 10 — Reporting Summary [file 41467_2023_38121_MOESM10_ESM.pdf]

nature portfolio

Corresponding author(s): Xiaohui Fan, Bing Zhang

Last updated by author(s): Mar 30, 2023

Reporting Summary

Nature Portfolio wishes to improve the reproducibility of the work that we publish. This form provides structure for consistency and transparency in reporting. For further information on Nature Portfolio policies, see our Editorial Policies and the Editorial Policy Checklist.

Statistics

For all statistical analyses, confirm that the following items are present in the figure legend, table legend, main text, or Methods section.

n/a	Confirmed	
☐	☒	The exact sample size (*n*) for each experimental group/condition, given as a discrete number and unit of measurement
☐	☒	A statement on whether measurements were taken from distinct samples or whether the same sample was measured repeatedly
☐	☒	The statistical test(s) used AND whether they are one- or two-sided<br>*Only common tests should be described solely by name; describe more complex techniques in the Methods section.*
☒	☐	A description of all covariates tested
☐	☒	A description of any assumptions or corrections, such as tests of normality and adjustment for multiple comparisons
☐	☒	A full description of the statistical parameters including central tendency (e.g. means) or other basic estimates (e.g. regression coefficient) AND variation (e.g. standard deviation) or associated estimates of uncertainty (e.g. confidence intervals)
☐	☒	For null hypothesis testing, the test statistic (e.g. *F*, *t*, *r*) with confidence intervals, effect sizes, degrees of freedom and *P* value noted<br>*Give P values as exact values whenever suitable.*
☒	☐	For Bayesian analysis, information on the choice of priors and Markov chain Monte Carlo settings
☒	☐	For hierarchical and complex designs, identification of the appropriate level for tests and full reporting of outcomes
☐	☒	Estimates of effect sizes (e.g. Cohen's *d*, Pearson's *r*), indicating how they were calculated

Our web collection on statistics for biologists contains articles on many of the points above.

Software and code

Policy information about availability of computer code

Data collection: No software was used for data collection

Data analysis: The scSpace package is available at https://github.com/ZJUFanLab/scSpace/.

Specific package version used for analysis are as follows (python 3.8.5):
numpy==1.23.4
pandas==1.5.0
scikit-learn==1.1.2
scipy==1.9.2
scanpy==1.9.1
igraph==0.10.2
leidenalg==0.9.0
tqdm==4.64.1
torch==1.12.1
SpaGCN==1.2.5
STAGATE==1.0.1

Specific package version used for analysis are as follows (R 4.1.0):
Seurat== 4.1.0
splatter==1.16.1
cgdsr==1.3.0

survival==3.2-13
msigdbr==7.4.1
fgsea==1.18.0
GSVA==1.44.5
limma==3.52.4
SpaTalk==1.0
BayesSpace==1.6.0
DR-SC==3.0
scCoGAPS==3.16.0

Specific web tools used for analysis are as follows:
Metascape==3.5
CIBERSORTx==1.0.5

For manuscripts utilizing custom algorithms or software that are central to the research but not yet described in published literature, software must be made available to editors and reviewers. We strongly encourage code deposition in a community repository (e.g. GitHub). See the Nature Portfolio guidelines for submitting code & software for further information.

Data

Policy information about availability of data

All manuscripts must include a data availability statement. This statement should provide the following information, where applicable:

- Accession codes, unique identifiers, or web links for publicly available datasets
- A description of any restrictions on data availability
- For clinical datasets or third party data, please ensure that the statement adheres to our policy

Data used in this study are downloaded from publicly available datasets. No experimental data conducted by ourselves are used.

(1) 10X Visium data of the human dorsolateral prefrontal cortex (DLPFC) [http://spatial.libd.org/spatialLIBD/];
(2) STARmap data of the mouse primary visual cortex V1 [https://www.dropbox.com/sh/f7ebheru1lbz91s/AABYSSjSTppBmVmWl2H4s_K-a?dl=0];
(3) “Spatial Transcriptomics” data of the human HER2 breast cancer [https://zenodo.org/record/5511763#.Y6kMduxBzUI];
(4) single-cell RNA-seq data and “Spatial Transcriptomics” data of the human skin squamous cell carcinoma (SCC): GEO accession: “GSE144240 [https://www.ncbi.nlm.nih.gov/geo/query/acc.cgi?acc=GSE144240]”;
(5) single-cell RNA-seq data of the mouse intestine: GEO accession: “GSE109413 [https://www.ncbi.nlm.nih.gov/geo/query/acc.cgi?acc=GSE109413]”;
(6) 10X Visium data of the mouse intestine: GEO accession: “GSE169749 [https://www.ncbi.nlm.nih.gov/geo/query/acc.cgi?acc=GSE169749]”;
(7) single-cell RNA-seq data of the mouse liver: GEO accession: “GSE84498 [https://www.ncbi.nlm.nih.gov/geo/query/acc.cgi?acc=GSE84498]”;
(8) 10X Visium data of the mouse liver [https://www.livercellatlas.org];
(9) single-cell RNA-seq data of the mouse neocortex [https://portal.brain-map.org/atlases-and-data/rnaseq/mouse-v1-and-alm-smart-seq];
(10) single-cell RNA-seq data of the mouse kidney: GEO accession: “GSE129798 [https://www.ncbi.nlm.nih.gov/geo/query/acc.cgi?acc=GSE129798]”; (
11) 10X Visium of the mouse kidney [https://www.10xgenomics.com/resources/datasets];
(12) Slide-seq v2 data of the mouse kidney [https://cellxgene.cziscience.com/collections/8e880741-bf9a-4c8e-9227-934204631d2a];
(13) single-cell ATAC-seq data of the mouse cortex: ArrayExpress: “E-MTAB-11264 [https://www.ebi.ac.uk/biostudies/arrayexpress/studies/E-MTAB-11264?query=E-MTAB-11264]”;
(14) single-cell RNA-seq data and “Spatial Transcriptomics” data of the human embryonic heart [https://data.mendeley.com/datasets/mbvhhf8m62/2];
(15) single-nucleus RNA-seq data of the middle temporal gyrus (MTG) of the human cortex [https://portal.brain-map.org/atlases-and-data/rnaseq/human-mtg-smart-seq];
(16) single-nucleus RNA-seq data of the multiple cortical areas (MTG, ACC, V1C, M1C, S1C and A1C) of the human cortex [https://portal.brain-map.org/atlases-and-data/rnaseq/human-multiple-cortical-areas-smart-seq];
(17) single-cell RNA-seq data of the human melanoma: GEO accession: “GSE72056 [https://www.ncbi.nlm.nih.gov/geo/query/acc.cgi?acc=GSE72056]”;
(18) “Spatial Transcriptomics” data of the human melanoma [https://www.spatialresearch.org/resources-published-datasets/doi-10-1158-0008-5472-can-18-0747/];
(19) single-cell RNA-seq data of the human lung of lethal COVID-19 [https://singlecell.broadinstitute.org/single_cell/study/SCP1219];
(20) 10X Visium of the normal human lung: GEO accession: “GSE178361 [https://www.ncbi.nlm.nih.gov/geo/query/acc.cgi?acc=GSE178361]”;
(21) GeoMx DSP targeted ST data of the human lung of COVID-19 [https://doi.org/10.5281/zenodo.4635285].

Human research participants

Policy information about studies involving human research participants and Sex and Gender in Research.

Reporting on sex and gender	Reporting on sex and gender is not relevant to our study.
Population characteristics	Population characteristics is not relevant to our study.
Recruitment	Recruitment is not relevant to our study.
Ethics oversight	Ethics oversight is not relevant to our study.

Note that full information on the approval of the study protocol must also be provided in the manuscript.

Field-specific reporting

Please select the one below that is the best fit for your research. If you are not sure, read the appropriate sections before making your selection.

☒ Life sciences ☐ Behavioural & social sciences ☐ Ecological, evolutionary & environmental sciences

For a reference copy of the document with all sections, see nature.com/documents/nr-reporting-summary-flat.pdf

Life sciences study design

All studies must disclose on these points even when the disclosure is negative.

Sample size	No data collection was involved in our study. We analyzed 14 publicly available datasets, including human dorsolateral prefrontal cortex, human HER2 breast cancer, human skin squamous cell carcinoma, mouse V1 neocortex, mouse intestine, mouse liver, mouse kidney, human developing heart, human cortex, human melanoma, and human lung in COVID-19.These 14 datasets represent data generated from a wide range of tissue types, and show that scSpace is able to maintain robustness with different types of scRNA-seq data.
Data exclusions	No data is excluded
Replication	All attempts at replication were successful and can be performed independently.
Randomization	The allocation was random involving the computational algorithms.
Blinding	Blinding is not relevant to our study because no new data collection was involved in the present study.

Reporting for specific materials, systems and methods

We require information from authors about some types of materials, experimental systems and methods used in many studies. Here, indicate whether each material, system or method listed is relevant to your study. If you are not sure if a list item applies to your research, read the appropriate section before selecting a response.

Materials & experimental systems

n/a	Involved in the study	
☒	☐	Antibodies
☒	☐	Eukaryotic cell lines
☒	☐	Palaeontology and archaeology
☒	☐	Animals and other organisms
☒	☐	Clinical data
☒	☐	Dual use research of concern

Methods

n/a	Involved in the study	
☒	☐	ChIP-seq
☒	☐	Flow cytometry
☒	☐	MRI-based neuroimaging
